# Dissecting the human *BDNF* locus: Bidirectional transcription, complex splicing, and multiple promoters^☆^

**DOI:** 10.1016/j.ygeno.2007.05.004

**Published:** 2007-09

**Authors:** Priit Pruunsild, Anna Kazantseva1, Tamara Aid, Kaia Palm, Tõnis Timmusk

**Affiliations:** Department of Gene Technology, Tallinn University of Technology, Akadeemia tee 15, Tallinn 19086, Estonia

**Keywords:** Neurotrophic factor, Brain, neuron, Alternative splicing, BDNF, Natural antisense transcript, RNA duplex, Lin-7c/Mals-3/veli3

## Abstract

Brain-derived neurotrophic factor (BDNF), a member of the nerve growth factor family of neurotrophins, has central roles in the development, physiology, and pathology of the nervous system. We have elucidated the structure of the human *BDNF* gene, identified alternative transcripts, and studied their expression in adult human tissues and brain regions. In addition, the transcription initiation sites for human *BDNF* transcripts were determined and the activities of *BDNF* promoters were analyzed in transient overexpression assays. Our results show that the human *BDNF* gene has 11 exons and nine functional promoters that are used tissue and brain-region specifically. Furthermore, noncoding natural antisense RNAs that display complex splicing and expression patterns are transcribed in the *BDNF* gene locus from the *antiBDNF* gene (approved gene symbol *BDNFOS*). We show that *BDNF* and *antiBDNF* transcripts form dsRNA duplexes in the brain in vivo, suggesting an important role for *antiBDNF* in regulating *BDNF* expression in human.

Brain-derived neurotrophic factor (BDNF) is a member of the nerve growth factor family of neurotrophins. During development BDNF supports the survival and differentiation of selected neuronal populations of the peripheral and central nervous systems and participates in axonal growth and pathfinding and in the modulation of dendritic growth and morphology [1,2]. BDNF also has a prominent role in later stages of nervous system development and in the adult by regulating synaptic transmission and plasticity and acts as a central modulator of pain [3]. BDNF has been shown to be a modulator of synaptogenesis in vivo [4] and has a functional role in the expression of LTP in the hippocampus [5]. Data accumulated in recent years suggest that neuronal activity regulates transcription of *BDNF,* transport of *BDNF* mRNA and protein into dendrites, and secretion of BDNF protein, which are important for the formation of appropriate synaptic connections and for learning and memory during development and in adults [6]. A single-nucleotide polymorphism in the human *BDNF* gene, resulting in a valine to methionine substitution (Val66Met) in the prodomain, has been shown to lead to reduced activity-induced BDNF secretion and memory impairment [7]. BDNF signaling has been shown to be critical in several neuropsychiatric and neurodegenerative diseases [1], for example, Huntington disease [8]. These results taken together show that BDNF has numerous important roles in brain development, physiology, and pathology.

During development, BDNF protein expression is more abundant in the nervous system compared to other tissues and its levels are dramatically increased in the brain during postnatal development [9]. In the adult nervous system, *BDNF* displays a widespread distribution pattern, with the highest levels of mRNA and protein in the hippocampus, amygdala, cerebral cortex, and hypothalamus [9–12]. *BDNF* mRNA expression is mostly confined to neurons and there are only a few brain areas where *BDNF* mRNA is not detected [10–12]. *BDNF* expression in adult tissues is detectable also outside of the central nervous system. Lower *BDNF* mRNA levels than in the hippocampus have been detected in the thymus, liver, spleen, heart, and lung [9,11,13,14].

The structure and regulation of the *BDNF* gene have been studied in rodents [15–17]. Mouse and rat *BDNF* genes have eight 5′ exons containing separate promoters upstream of each exon and one 3′ exon encoding the mature BDNF protein [17]. Multiple promoters determine tissue-specific expression of the *BDNF* transcripts [15,17]. The human *BDNF* has also been shown to consist of multiple 5′ noncoding exons and one coding exon, which give rise to alternatively spliced transcripts [18–20]. According to the most recent description of the organization of the human *BDNF* gene locus and alternatively spliced transcripts the human *BDNF* has seven noncoding and one coding exon [19]. Provided that only a partial description of the transcripts was given and expression of the alternative transcripts was studied in only a few brain regions and was not investigated in human nonneural tissues, we undertook the current study to analyze thoroughly the structure of the human *BDNF* gene, characterize the expression of alternatively spliced *BDNF* mRNAs in different human tissues and brain regions, and identify and study the activities of alternative human *BDNF* promoters.

## Results

### Structure and alternative splicing of human BDNF and antiBDNF (approved gene symbol BDNFOS)

To reexamine the human *BDNF* gene structure and identify mRNAs transcribed from the gene, in silico analysis, 5′ rapid amplification of cDNA ends (5′ RACE), and RT-PCR analyses were performed. First, all *BDNF* mRNAs and expressed sequence tags (ESTs) available at the NCBI database (http://www.ncbi.nlm.nih.gov) were analyzed. Primers designed for PCR analyses are presented in Supplementary Table 1. Total RNAs of the adult human frontal cerebral cortex, medulla, and hippocampus were used as templates in the RT-PCR. Second, to identify novel *BDNF* transcripts and to determine the transcription start sites for the human *BDNF* transcripts, 5′ RACE of human adult hippocampal and cerebellar RNA was performed using antisense primers specific for the 3′ exon and for the 5′ exons (Supplementary Table 1).

**Fig. 1:**
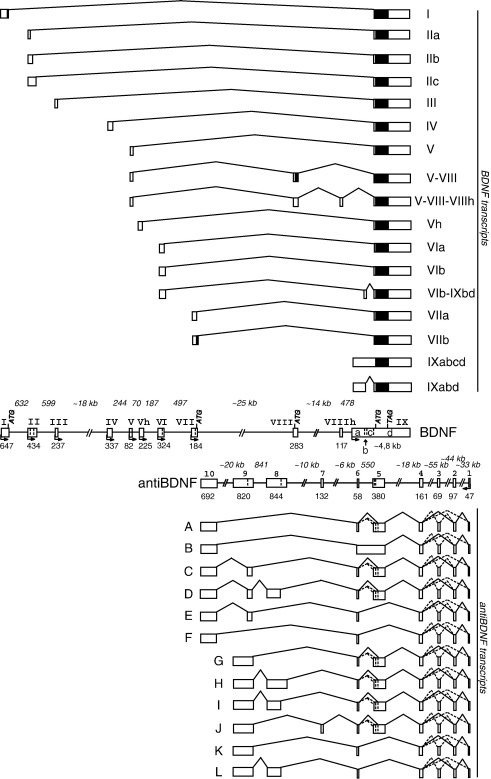
Structure and alternative transcripts of the human *BDNF* (top) and *antiBDNF* (bottom) genes. The structural organization of the exons and introns was determined by analyzing genomic and mRNA sequence data using bioinformatics, RT-PCR, and 5′ RACE. Exons are shown as boxes and introns as lines. Filled boxes and open boxes indicate the translated regions of the exons and the untranslated regions of the exons, respectively. The numbers below the exons and above the introns indicate their sizes. Exon and intron sizes are in base pairs, if not indicated otherwise. Arrows indicate the transcription start sites. ATG and TAG mark the positions of the translational start and stop codons, respectively. Vertical dashed lines indicate alternative splicing sites for the respective exons. *BDNF* exon IX is divided into regions “a”, “b”, “c”, and “d” as indicated in the box marking the position of exon IX. *BDNF* transcript names relate to the upstream exons used in front of the major 3′ exon IXd. “A”–“L” mark *antiBDNF* transcripts. Solid lines connecting the exons of transcripts represent the major splicing patterns of exons. Dashed lines connecting the exons of transcripts represent the minor splicing patterns of *antiBDNF*. Exon numbers are shown in Roman numerals for the *BDNF* gene and in Arabic numerals for the *antiBDNF* gene.

**Fig. 2:**
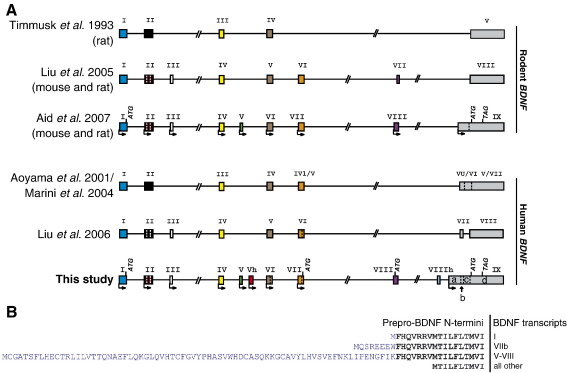
(A) Comparison of the human and rodent *BDNF* gene structures proposed by different studies. Structures presented are according to Timmusk et al. [15], Liu et al. [16], Aid et al. [17], Aoyama et al. [20], Marini et al. [40], and Liu et al. [19] and the human *BDNF* gene structure determined in this study. Exons are shown as boxes and introns as lines. The identical human exons and the rodent exons homologous to the human exons are shown in the same color in all the structures. Novel exons determined by this study are in green (V), red (Vh), purple (VIII), and light blue (VIIIh). (B) Amino acid sequences of different potential prepro-BDNF N-termini. Amino acids encoded by exon IX are in black and sequences encoded by alternative 5′ exons are in blue. The transcripts encoding the respective N-termini of BDNF are listed adjacent to the N-terminal sequences.

*BDNF* gene exon–intron boundaries and genomic locations were determined by BLAT algorithm (http://genome.ucsc.edu/cgi-bin/hgBlat) and by direct comparison of PCR-amplified sequences with genomic DNA sequence from the NCBI database (Accession No. AF411339). The designation of the human *BDNF* exons in this study is consistent with the naming of the mouse and rat *BDNF* exons described in the study by Aid et al. [17]. The exons unique to human *BDNF* are marked with the letter “h” and are named with the same number as the neighboring upstream exon. Our analysis showed that the human *BDNF* gene spans ∼ 70 kb and consists of 11 exons (Fig. 1). *hBDNF* exons named by Liu et al. [19] are designated here as old exons. Comparison of our data with that of Liu et al. [19] shows that exons I–IV correspond to the respective old exons; exons V, Vh, VIII, and VIIIh are novel exons; exons VI and VII correspond to old exons V and VI, respectively; and exon IX variants IXb and IXd correspond to old exons VII and VIII, respectively (Fig. 2A). Nine of the exons, I, II, III, IV, V, Vh, VI, VII, and IX, can be defined as 5′ exons (Fig. 1). Cloning and sequencing of the 5′ RACE products revealed that the transcription start sites relative to the 3′ end of the respective exon are located as follows: − 647 and − 428 nt for exon I; − 433, − 423, − 422, − 416, − 407, − 400, − 226, − 224, − 204, − 200, − 78, and − 47 nt for exon II; − 237 and − 191 nt for exon III; − 337, − 333, − 274, and − 215 nt for exon IV; − 82, − 80, and − 79 nt for exon V; − 225 and − 222 nt for exon Vh; − 324; − 323, − 319, − 318, and − 315 nt for exon VI; and − 184 nt for exon VII (Supplementary Fig. 1). We determined that human *BDNF* transcription starts also from the last exon, exon IX, − 1102 nt upstream of the translation start site in this exon (Supplementary Fig. 1). No major differences in the transcription start site locations were observed when 5′ RACE products from hippocampal or cerebellar RNA were analyzed. Two exons, VIII and VIIIh, are rarely used and always in combination with exon V as the 5′ exon (Fig. 1). Exons II, III, IV, V, Vh, VI, and VIIIh are untranslated exons and translation of the transcripts containing these exons starts from the ATG positioned in exon IX (Supplementary Fig. 1). Exons I, VII, and VIII contain in-frame ATG codons that could be used as translation start sites leading to the prepro-BDNF proteins with longer N-termini (Fig. 2B).

In addition, we identified alternative splice donor sites in exons II, V, and VI (Fig. 1, Supplementary Fig. 1, and Table 2). Usage of these splice sites leads to the formation of transcripts with different 5′ UTR lengths but does not affect the coding region of *BDNF.* Characterization of the exon–intron boundary sequences showed that not all exon–intron splice junctions adhere to the GU–AG rule characteristic of eukaryotes. We found that exon VII is unique because the splice donor site used contains nucleotides GG instead of the conventional GU sequence (Supplementary Table 2). Exon IX, which encodes the BDNF protein and 3′ UTR, is subjected to internal splicing and/or transcription initiation upstream of exon IX that leads to the generation of alternative transcripts containing variants of exon IX. These exon IX variants comprise different regions or combinations of regions of exon IX that were designated “a”, “b”, “c”, and “d” (Fig. 1). Exon IX is used mostly in conjunction with the upstream exons (I–VIII, VIIIh), and in that case only the most 3′ region of exon IX, IXd, is included in the mature transcripts. On rare occasions when exon VI is the 5′ exon, alternative splicing occurs within exon IX leading to the inclusion of two regions, IXb and IXd, in the mRNAs. When transcription is initiated upstream of exon IX the transcripts are not subjected to internal splicing and contain all the regions of exon IX: IXa, IXb, IXc, and IXd. In extremely rare cases exon IX region “c” is spliced out (Fig. 1 and Supplementary Fig. 1).

Recently it was reported that natural antisense transcripts are transcribed from the human *BDNF* gene locus [19]. We analyzed the exon–intron junctions of amplified *BDNF* cDNAs and also noticed that several mRNAs are transcribed in an antisense direction compared to BDNF mRNAs. This finding was also confirmed by orientation-specific RT-PCR and by RNase protection assay (data not shown). We named the gene and transcripts transcribed from the opposite strand of *BDNF* as *antiBDNF* (for antisense *BDNF;* part of it is described by Liu and colleagues and designated as OSBDNF—Liu et al. [19]). The *antiBDNF* gene spans ∼ 191 kb and consists of at least 10 exons (exons 1–10) and is transcribed from one promoter as shown by our 5′ RACE analyses (Fig. 1). All intron–exon boundaries of the *antiBDNF* gene are consistent with the GU–AG consensus (Supplementary Table 2).

**Supplementary Figure 2:**
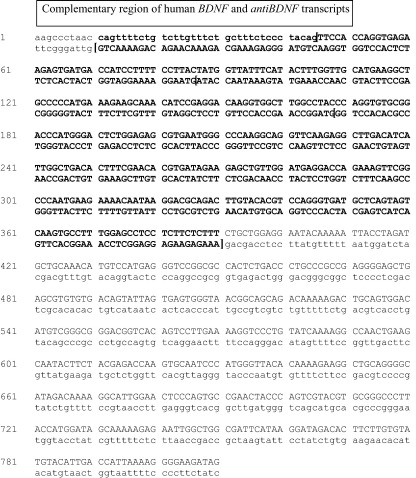


Exons 1–4 of the *antiBDNF* gene are located downstream of the *BDNF* gene (Fig. 1). Exon 5, 345 bp in length, overlaps regions IXc and IXd and exon 6 overlaps region IXa of the *BDNF* coding exon. Exons 7–10 of *antiBDNF* are located in the introns of *BDNF*. In silico and RT-PCR analyses showed that alternative splicing from the *antiBDNF* pre-mRNA produces more than 300 transcripts, but exon 1 of *antiBDNF* is always used as the most 5′ exon for all the transcripts. Of note, our bioinformatics analysis showed that *antiBDNF* exon 1 is in head-to-head orientation with exon 1 of *Lin-7c/Mals-3/veli3,* suggesting that a bidirectional promoter controls the expression of these genes. The majority of the *antiBDNF* alternative transcripts contain exons 5 and 6, which are complementary to the *BDNF* protein-coding exon IX. However, in several *antiBDNF* transcripts exon 5 is skipped out. In addition, exon 5 of *antiBDNF* could be spliced using three alternative splice donor sites (Fig. 1, Supplementary Fig. 2), and the lengths of exons 8 and 9 can vary because of usage of internal alternative splice acceptor sites (Fig. 1). There are no potential open reading frames in any of the identified mRNAs transcribed from the *antiBDNF* gene, suggesting that these transcripts are non-protein-coding, as proposed also by Liu et al. [19].

### Expression of alternatively spliced BDNF and antiBDNF mRNAs in adult human tissues

**Fig. 3:**
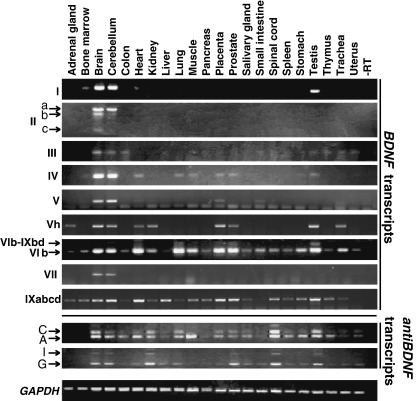
Semiquantitative analysis of human *BDNF, antiBDNF,* and control *GAPDH* mRNA expression in adult human tissues by RT-PCR. Roman numerals on the left indicate the detected *BDNF* transcripts and the 5′ exon-specific primers used in combination with an antisense primer located in the *BDNF* coding region in exon IXd (Supplementary Table 1). A, C, G, and I refer to the respective *antiBDNF* transcripts shown in Fig. 1.

Expression of *BDNF* and *antiBDNF* transcripts was determined by RT-PCR in 22 different adult human tissues (Fig. 3). The results showed that human *BDNF* alternative transcripts are expressed in a tissue-specific manner. The levels of the majority of the human *BDNF* transcripts were highest in the brain. However, several alternative *BDNF* mRNAs showed relatively high expression levels in nonneural tissues. For example, expression levels of transcripts containing exons VI and IXabcd were high in the heart, placenta, and prostate. Transcripts containing exons I, Vh, VI, and IXabcd were highly expressed in the testis. High levels of transcripts containing exon VI were expressed also in the lung. Several *BDNF* mRNAs were expressed at moderate or low levels in the adrenal gland (exon Vh and exon IXabcd transcripts), bone marrow (exons I, VI, and IXabcd transcripts), kidney, muscle, stomach, spinal cord (exons Vh and VI transcripts), liver (exon IXabcd transcripts), small intestine (exon VI transcripts), and trachea (exons Vh, VI, and IXabcd transcripts). Low levels of exon IXabd transcripts were observed only in some cell lines (data not shown). *BDNF* mRNAs containing exons II and VII were expressed exclusively in the brain. Altogether, the results indicate that transcripts containing exons II, III, IV, V, and VII are predominantly brain-specific. Transcripts containing exons I and Vh are, in addition to brain, expressed in certain peripheral tissues, and transcripts containing exons VI and IXabcd show a wide pattern of expression.

*antiBDNF* transcripts were present at different levels in almost all human tissues analyzed (Fig. 3). High expression of human *antiBDNF* transcripts was detected in the brain, kidney, spinal cord, and testis. Moderate levels of *antiBDNF* RNA were seen in the lung, prostate, salivary gland, spleen, stomach, and uterus. Low *antiBDNF* expression levels were detected in the adrenal gland, liver, placenta, small intestine, and trachea. Certain alternative transcripts of *antiBDNF* were expressed in a tissue-specific manner. For example, *antiBDNF* transcripts with exon 10 were present in the colon and muscle, whereas transcripts with exon 9 were not expressed in these tissues. Taken together, *BDNF* and *antiBDNF* expression patterns were distinct, although partially overlapping.

### Expression of alternatively spliced BDNF and antiBDNF mRNAs in adult human brain regions

**Fig. 4:**
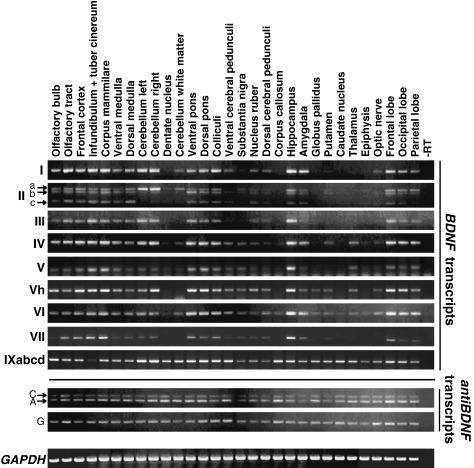
Semiquantitative analysis of human *BDNF, antiBDNF,* and control *GAPDH* mRNA expression by RT-PCR in different human brain regions. Roman numerals on the left indicate the detected BDNF transcripts and the 5′-exon-specific primers used in combination with an antisense primer located in the *BDNF* coding region in exon IXd (Supplementary Table 1). A, C, and G refer to the respective *antiBDNF* transcripts shown in Fig. 1.

Expression analysis of human *BDNF* and *antiBDNF* transcripts in 30 different adult brain regions was performed by RT-PCR (Fig. 4). Several differences in the expression of alternatively spliced human *BDNF* transcripts were detected. The results showed that all *BDNF* transcripts were expressed at high levels in the corpus mammillare (mammillary body), pons, hippocampus, frontal cortex, colliculi, and olfactory tract. All *BDNF* transcripts except the ones containing exons V and VII were expressed at high levels in the cerebellum and medulla, and all transcripts but those containing exon IXabcd were expressed at high levels in the infundibulum. *BDNF* expression in the dentate nucleus, white matter of the cerebellum, substantia nigra, nucleus ruber (red nucleus), and epiphysis was very low. *BDNF* expression was also very low in the globus pallidus, striatum (caudate nucleus and putamen), and thalamus, with the exception of exon IXabcd transcripts, which were expressed at relatively high levels in these regions. In the amygdala only transcripts containing exons I, IV, and VI were expressed at high levels. In the corpus callosum only exon VI and IXabcd transcripts were detected. Notably, comparison of expression levels of individual transcripts in different brain areas indicated that *BDNF* exon II transcript levels were much higher in the cerebellum than in other brain areas. Exon IXabcd mRNAs were expressed at relatively similar levels in all brain regions, with only infundibulum having very low expression levels. Interestingly, in the brain structures that contain only glial cells and axons and do not contain neuronal cell bodies, such as corpus callosum and optic nerve, exon IXabcd transcripts were predominantly detected. Transcripts containing exons I, Vh, and VI were also present in the optic nerve, although at low levels. *antiBDNF* transcripts were expressed in all studied brain structures at similar levels (Fig. 4).

### Promoter activities of the 5′ flanking regions of human BDNF and antiBDNF upstream exons

Since the promoter regions of the human *BDNF* gene have not been analyzed previously and hypothesizing that a functional promoter precedes each of the identified 5′ exon of *BDNF* and that there is a promoter upstream of *antiBDNF* exon 1, the activities of nine potential promoter regions within the *BDNF* gene (namely the upstream sequences of exons I, II, III, IV, V, Vh, VI, VII, and IXabcd) and the region upstream of exon 1 of *antiBDNF* were analyzed for transcription-promoting activity using chloramphenicol acetyltransferase (CAT) assays. The putative promoter regions, each ∼ 0.2–1.3 kb in length and containing a part of the respective 5′ UTR and 5′ flanking genomic sequence (Supplementary Fig. 1), were isolated and cloned into the pBLCAT2 vector in front of the *CAT* gene. The promoter constructs were transfected into human embryonic kidney HEK293T and mouse neuroblastoma N2a cells and the promoter activities were analyzed.

**Fig. 5:**
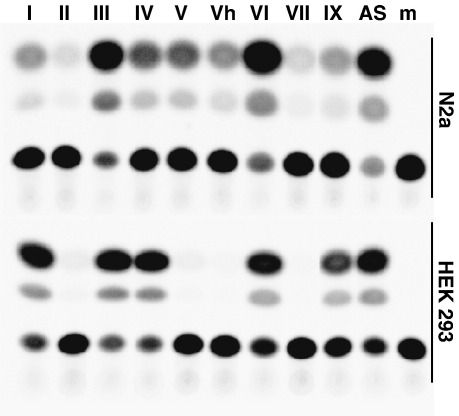
Analyses of *BDNF* and *antiBDNF* promoter activities in HEK293T and N2a cells. The relative activities of the 5′ flanking regions of *BDNF* exons I, II, III, IV, V, Vh, VI, VII, and IXabcd and *antiBDNF* to promote CAT expression are shown. The promoter regions cloned in front of the *CAT* gene are shown in Supplementary Fig. 1. Note that the activities of *BDNF* promoters II, V, Vh, and VII in HEK239T cells were detectable using longer reaction times. m, mock-transfected cells, negative control.

The results showed that all the regions upstream of the 5′ exons of the *BDNF* gene and exon 1 of the *antiBDNF* gene were functional and could activate CAT expression (Fig. 5). Thus it was concluded that the regions upstream of the 5′ exons of the *BDNF* gene and exon 1 of the *antiBDNF* gene act as separate promoters. However, the activities of the promoters varied and differences were detected also between the cell lines used. The activities of promoters upstream of exons II, V, Vh, and VII were somewhat lower compared to other promoters in N2a cells. In HEK293T cells the activities of these promoters could be detected only after longer reaction times. Other promoters showed similar activities in both of the cell lines with *BDNF* promoters upstream of exons III and VI and the *antiBDNF* promoter being the strongest in both cell lines. However, promoters upstream *BDNF* exons I, IV, and IXabcd were slightly (about twofold) more active in HEK293T cells than in N2a cells.

### Human BDNF and antiBDNF transcripts form RNA duplexes in adult human brain in vivo

**Fig. 6:**
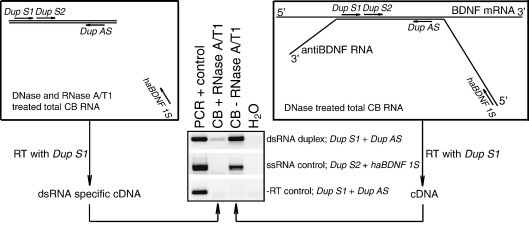
*BDNF* and *antiBDNF* transcripts form dsRNA duplexes in the human brain in vivo. A schematic representation of the RNA duplex detection assay is shown. Briefly, total human cerebellar RNA was DNase treated. This RNA was divided into two—one half was treated with RNase A/T1 (box on the left) and the other was used as a − RNase control (box on the right). Both RNAs were reverse transcribed (RT) with a *BDNF*/*antiBDNF* complementary region-specific primer, Dup_S1. Subsequently these cDNAs were used as templates in PCR to detect *BDNF*/*antiBDNF* duplex with primers Dup_S1 and Dup_AS. ssRNA contamination control reaction was conducted with primers Dup_S2 and haBDNF_S1. The − RT reaction was used for detection of genomic DNA contamination using primers Dup_S1 and Dup_AS. Lines indicate RNAs, double line marks the complementary region of *BDNF* exon IXd and *antiBDNF* exon 5. Primer positions are indicated with arrows parallel with the lines and primer names are in italic. Human hippocampal cDNA synthesized using an oligo(dT) primer (PCR + control) was used as positive control for all the reactions.

According to our data human *BDNF* and *antiBDNF* are coexpressed in many tissues studied. The complementary region of the majority of spliced *BDNF* and *antiBDNF* transcripts spans 222 nt or more depending on the splicing donor site used for *antiBDNF* exon 5 (Supplementary Fig. 2 and Supplementary Table 2). Based on this knowledge we hypothesized that if *antiBDNF* has a regulatory role in *BDNF* expression, the complementary RNAs might form RNA–RNA duplexes in vivo. To study this hypothesis we performed a PCR-based assay. Briefly, RNase A/T1-treated RNA from adult human cerebellum was used as a potential double-stranded RNA (dsRNA) template for cDNA synthesis and the existence of the duplexes was analyzed by PCR with primers specific for the complementary region of *BDNF* and *antiBDNF* (Supplementary Table 1). Our results showed that *BDNF*/*antiBDNF* dsRNA duplexes are present in the human brain in vivo (Fig. 6). Control experiments using a primer targeting the region of *antiBDNF* RNA outside of the complementary sequence in combination with a primer specific for the complementary region and experiments using RNA template in which the reverse transcription reaction was omitted showed that the RNA duplex-specific product was not the result of single-strand RNA (ssRNA) or genomic DNA contamination, respectively.

## Discussion

Previous studies have revealed that the human *BDNF* gene consists of seven putative 5′ exons and one protein-coding exon [19,20]. However, the expression patterns of different exons have not been thoroughly studied and possible linkage of these exons to separate promoters has not been investigated. Here we show that the human *BDNF* gene, extending over 70 kb, contains 11 exons. The 3′ exon encodes all or most of the protein depending on the 5′ exon used. Independent of the 5′ exon usage, two separate polyadenylation signals in exon IX can be utilized in *BDNF* transcripts. In addition, our data showed that the human *BDNF* gene comprises nine functional promoters.

The structures of the human *BDNF* gene and transcripts determined in this study are in good agreement with the results obtained for the rat and mouse *BDNF* genes [17]. Some differences are present, though. First, human *BDNF* contains two more exons than rodent *BDNF.* Compared to the rat and mouse genes [17] there is an additional exon, exon Vh, linked to a promoter between exons V and VI. Human *BDNF* exon VIIIh, which is not linked to a separate promoter, is also not present in rodent *BDNF.* Furthermore, cryptic splicing donor and acceptor sites are used in human exon IX leading to transcripts containing exons IXbd and IXabd. These transcripts have not been detected in rodents [17]. All this adds more complexity to the regulation of human *BDNF.* Second, in most cases, the usage of alternative promoters in the human *BDNF* gene leads to the expression of transcripts with different 5′ UTRs and with the protein-coding region in the common 3′ exon IXd. However, usage of an alternative upstream in-frame translation start site containing exon I, VII, or VIII could potentially lead to human BDNF prepro-proteins with longer N-termini. Only the translation initiation codon within exon I is characteristic of rodent *BDNF* genes, suggesting that there are more BDNF protein isoforms in human than in rodents. Third, although the transcription initiation sites are generally in good agreement with the respective regions in rodents, we identified more transcription start sites for human exons II and IV than had been reported previously for the rodent *BDNF* respective exons. Fourth, in contrast to the rodent *BDNF* genes we found that exon VIII of the human *BDNF* is not used as a 5′ exon as determined by the 5′ RACE analysis. We show that in human the rarely used exon VIII of *BDNF* is exclusively spliced to exon V. Exon V can also be spliced to exon IXd without including exon VIII. Exon VIII was not detected in any transcript other than the ones starting with exon V, pointing to a possible functional regulation between the usage of a certain promoter and subsequent splicing. Similar promoter-governed splicing regulation has been identified for the human nitric oxide synthase (*NOS1*) [21] and mouse *bcl-X* [22] genes, for example. This kind of splicing regulation is especially interesting provided the notion that exon VIII of *BDNF* contains one of the alternative ATGs that may lead to the synthesis of a prepro-BDNF protein with an alternative N-terminus.

We analyzed the splicing of the human *BDNF* pre-mRNAs and expression of consequent alternative mRNAs in detail. We found that *BDNF* transcripts containing exons II, III, IV, V, and VII are mostly brain-specific, whereas other *BDNF* mRNAs are also expressed at variable levels in nonneural tissues. Similar to the expression pattern of *BDNF* mRNAs in rodents [16,17], the most abundant transcripts in human nonneural tissues were transcripts containing exons VI and IXabcd that were expressed at high levels in several tissues, particularly heart, lung, skeletal muscle, testis, prostate, and placenta. To the best of our knowledge, these are the first data about the expression of alternatively spliced *BDNF* mRNAs in human nonneural tissues. In the human brain, expression of BDNF has been studied at the protein level using many different antibodies, the specificity of which is not always clear [23]. Fewer data are available about *BDNF* mRNA expression. In most studies on human *BDNF,* mRNA expression has been studied in only some regions of brain using postmortem tissue [23–25]. In two studies the expression of human *BDNF* mRNAs with alternative 5′ exons was examined in a few adult brain regions using RT-PCR [19,26]. Our study is the first to examine *BDNF* exon-specific mRNA levels across the whole human brain, thus adding important new data to *BDNF* expression in adult human brain. In the adult human brain, high levels of *BDNF* mRNAs were present in the hippocampus, cerebral cortex, amygdala, and cerebellum, which is similar to the previously reported data on the *BDNF* expression in rodent brain [10,11,15,27,28]. *BDNF* is expressed predominantly in neurons, although some studies have identified *BDNF* expression also in rodent astrocytes [29,30], microglia [31], and oligodendrocytes [32], both in vivo and in vitro. Here we show that some of the alternatively spliced human *BDNF* mRNAs, particularly transcripts containing exons VI and IXabcd, are present in vivo in the corpus callosum and optic nerve containing mostly oligodendroglial cells and axonal projections.

Gene expression in eukaryotes is a highly coordinated process involving regulation at many different levels, among which the regulation of transcription is one of the most important. Several types of *cis*-acting DNA sequence elements, including promoters, contribute to this process. About 18% of human genes have multiple promoters, which regulate and increase their transcriptional and translational potential [33]. Human *BDNF* promoter IV is the only promoter of the human *BDNF* gene that had been characterized so far [18]. In this study we show that *BDNF* gene expression is under the control of at least nine alternative tissue-specific promoters linked to separate 5′ exons. Alternative promoters of the *BDNF* gene could also be involved in developmental stage-specific expression and cell-type-specific expression, giving additional flexibility to the control of *BDNF* expression. Therefore, the data presented in this study show that the expression of the human *BDNF* gene is highly regulated at the level of transcription.

One of the results of our study was the characterization of endogenous noncoding antisense RNAs transcribed from the human *BDNF* gene locus. According to our data the *antiBDNF* gene consists of 10 exons and one functional promoter upstream of exon 1. We show that *antiBDNF* transcripts are expressed in almost all adult human tissues analyzed. High levels of *antiBDNF* mRNAs are present in the brain, kidney, spinal cord, and testis. Expression levels are low in adrenal gland, bone marrow, pancreas, small intestine, uterus, and some other tissues. In the adult brain, all *antiBDNF* transcripts are expressed at similar levels in all brain regions analyzed. We found that hundreds of different noncoding RNAs might be generated from the *antiBDNF* gene as a result of alternative splicing. Alternatively spliced isoform diversity is common to many eukaryotic organisms and it is particularly widely used in the nervous system [34]. Interestingly, *antiBDNF* is not present in rodents [16,17]. *antiBDNF* ESTs are also not available for chimpanzee and rhesus monkey although highly homologous sequences are present in the genomes of these animals (data not shown). All this suggests that *antiBDNF* could have evolved during primate/hominid evolution, as was proposed also by Liu et al. [19].

In this study we have shown that in the human brain *BDNF* and *antiBDNF* transcripts form dsRNA duplexes in vivo. This indicates that *antiBDNF* transcripts could have an important role in the regulation of *BDNF* expression in human. Several studies have shown that natural antisense transcripts (NATs) are involved in the regulation of gene expression in eukaryotes [35,36]. For example, NATs have been suggested to play an important role in the regulation of several genes encoding transcription factors that are important in eye development and function in mice [37]. Characterization of overlapping transcripts in various species indicates that this form of RNA-mediated gene regulation represents a widespread phenomenon [36,38]. NATs are particularly prevalent in the nervous system where they regulate the expression of several genes [35]. In the case of human *BDNF* and *antiBDNF,* the transcripts could act as *cis*-antisense RNAs and generate siRNAs targeting one of the initial transcripts, as do the natural *cis*-siRNAs described for genes involved in salt tolerance in *Arabidopsis thaliana* [39]. Other possible regulatory functions of *antiBDNF* would be direct inhibition of *BDNF* transcription or translation and/or regulation of *BDNF* pre-mRNA splicing. Our results show that the expression of *antiBDNF* and *BDNF* transcripts in different tissues is not mutually exclusive and that the levels of *BDNF* mRNA do not appear to be specifically reduced in tissues that express high levels of *antiBDNF* transcripts. However, it is possible that the *antiBDNF* transcripts could modulate the levels of *BDNF* provided they are coexpressed in the same cell.

In conclusion, this detailed characterization of the human *BDNF* gene locus opens up insights into the mechanisms governing *BDNF* gene regulation in human.

## Materials and methods

### RNA isolation, RT-PCR, and cloning and sequencing of RT-PCR products

Total RNAs from 23 human tissues were obtained from Clontech. Total RNAs from postmortem adult human brain regions were isolated using the RNAwiz RNA isolation reagent and treated with DNase (Ambion, USA) according to the supplier's protocol. All experiments with human tissues were approved by the local ethical committee. Five micrograms of total RNA was reverse-transcribed to cDNA with an oligo(dT) primer (Proligo, France) and SuperScript III reverse transcriptase using the SuperScript III First-Strand Synthesis System (Invitrogen, USA). PCR amplification was carried out using HOT FIREPol DNA polymerase (Solis Biodyne, Estonia), according to the manufacturer's instructions. One-fortieth of the first-strand cDNA reaction mix was used in the PCR. The exon-specific PCR primers were designed based on the sequence of the human *BDNF* gene (NCBI Accession No. AF411339), ESTs, and mRNA sequences from GenBank. Sequences for all primers are listed in Table 1 of the supplementary material. The lengths of the PCR products using the primer hBDNF_IXbAS in combination with the following primers were hBDNF_IS, 472 bp; hBDNF_IIS, 610, 527, and 312 bp; hBDNF_IIIS, 347 bp; hBDNF_IVS, 412 bp; hBDNF_VS, 673, 556, and 273 bp; hBDNF_VhS, 340 bp; hBDNF_VIS, 494, 387, and 369 bp; hBDNF_VIIS, 429 and 328 bp; and hBDNF_IXS, 597 and 363 bp. The lengths of the longest PCR products with haBDNF_1S in combination with the following primers were haBDNF_9AS, 947 bp, and haBDNF_10AS, 1483 bp. All products from the RT-PCR were cloned into the pCRII-TOPO vector (Invitrogen) and sequenced.

### Analyses of transcription start sites

The transcription start sites for the *BDNF* and *antiBDNF* transcripts were detected with 5′ RACE using the GeneRacer Kit (Invitrogen) for full-length, RNA ligase-mediated rapid amplification of 5′ cDNA ends, according to the manufacturer's instructions. Briefly, 5 μg of total RNA from human hippocampus and cerebellum was dephosphorylated and decapped. The GeneRacer RNA oligo was ligated to the decapped 5′ ends of the full-length mRNAs and reverse transcription of the mRNAs was performed. RACE-ready cDNAs were used as templates for subsequent PCR using the GeneRacer 5′ primer in combination with *BDNF* exon-specific primers (Supplementary Table 1). The PCR products were gel purified and cloned into the pCRII-TOPO (Invitrogen) vector and verified by sequencing.

### BDNF promoter–CAT reporter plasmids and CAT assay

PCR was performed to amplify promoter fragments of the human *BDNF* and *antiBDNF* genes with the appropriate primers (Supplementary Table 1). Human genomic DNA was used as a template. The amplified fragments were cloned into the pBL-CAT2 plasmid upstream of the coding region of the *CAT* gene, replacing the thymidine kinase promoter, and verified by sequencing. HEK293T and N2a cells were used for the analysis of promoter activities. The cells were maintained in Dulbecco's modified Eagle's medium supplemented with 10% fetal calf serum at 37°C in a 5% CO_2_ atmosphere. Transfection of cells with the promoter–CAT reporter plasmids was performed with FuGENE 6 (Roche Diagnostics, USA) according to the supplier's instructions. The transfected cells were harvested 40 h after transfection. The samples were incubated with ^14^C-labeled chloramphenicol and acetyl-CoA and the radioactive products were separated by thin-layer chromatography silica gel (Merck, USA) and visualized by autoradiography as described before [15].

### BDNF/antiBDNF RNA duplex analyses

Ten micrograms of human cerebellar RNA, isolated with RNAwiz (Ambion), was treated with DNase (Ambion Turbo DNA-*free*) for 30 min at 37°C according to the manufacturer's instructions, precipitated, and treated with RNase A/T1 (Ambion) for 30 min at 37°C in RNase buffer (300 mM NaCl, 10 mM Tris, pH 7.4, and 5 mM EDTA). The reaction was terminated with 0.4 mg/ml proteinase K (Roche) in 200 mM Tris, pH 7.4, 25 mM EDTA, and 1% SDS for 30 min at 37°C, and the dsRNA was phenol/chloroform extracted. cDNA was synthesized from this dsRNA using the *BDNF/antiBDNF* complementary region-specific primer Dup_S1 (Supplementary Table 1) with Superscript III (Invitrogen) according to the manufacturer's instructions in the presence of 5% DMSO. In subsequent RNA duplex detection PCR 1/20 of the cDNA was used along with primers specific for the *BDNF/antiBDNF* complementary region, Dup_S1 and Dup_AS (Supplementary Table 1; product size 156 bp). Primer haBDNF_1S recognizing *antiBDNF* exon 1 in combination with Dup_S2 (Supplementary Table 1; product size 490 bp) was used for detection of ssRNA contamination. In addition, a − RT reaction was used for detection of genomic DNA contamination using primers Dup_S1 and Dup_AS. All PCRs were performed as follows: 40 cycles of 94°C for 30 s, 57°C for 30 s, and 72°C for 30 s.
