# Supplementary material for: Dissecting the human BDNF locus: Bidirectional transcription, complex splicing, and multiple promoters
Source: Genomics. 2007 Sep;90(3):397–406. doi: 10.1016/j.ygeno.2007.05.004 (PMC2568880; doi:10.1016/j.ygeno.2007.05.004)
Supplement: Supplementary Table 1 [file mmc1.doc]

# Supplementary Table 1. Primers used in the study

| **Forward primers** |  |  |
| --- | --- | --- |
| **Name** | **Location** | **Sequence** |
| *hBDNF_IS* | exon I | GATGCCAGTTGCTTTGTCTTCTGTAG |
| *hBDNF_IIS* | exon II | GGGCGATAGGAGTCCATTCAGCACC |
| *hBDNF_IIIS* | exon III | AGTTTCGGGCGCTGGCTTAGAG |
| *hBDNF_IVS* | exon IV | GCTGCAGAACAGAAGGAGTACA |
| *hBDNF_VS* | exonV | TCGCGTTCGCAAGCTCCGTAGTG |
| *hBDNF_VhS* | exon Vh | GGCTGGAACACCCCTCGAA |
| *hBDNF_VIS* | exon VI | GGCTTTAATGAGACACCCACCGC |
| *hBDNF_VIIS* | exon VII | GAACTGAAAGGGTCTGCGACACTCT |
| *hBDNF_IXS* | exon IX | TTTCTCGTGACAGCATGAGCAG |
| *haBDNF_1S* | exon 1 | CGAGATCAGGAAGGTGGCCGAGT |
| *Prom_IS* | promoter I | TGCGCACCGGGGCTGTTAACTCAC |
| *Prom_IIS* | promoter II | TGCTTGTCTCTCAGCAGTCTTGC |
| *Prom_IIIS* | promoter III | AGAATCAGGCGGTGGAGGTGGTGTG |
| *Prom_IVS* | promoter IV | TGTGTTTGCTGGGGCTGGAAGTG |
| *Prom_VS* | exon IV | AGAGTGTGGGAGTTTTGGGGCCGA |
| *Prom_VIIS* | promoter VII | AGGGACGATTTGGAGTTACTCTTGG |
| *Prom_IX* | promoter IXa | CAGCCATTTCTGCATTCTGACC |
| *Prom_1S* | promoter 1 | GAATTGTACCGTGTGTCCATTCTC |
| *Dup_S1* | exon IX | CATGGGACTCTGGAGAGCGTGAAT |
| *Dup_S2* | exon IX | CGTGATAGAAGAGCTGTTGGATGAGG |
| **Reverse primers** |  |  |
| **Name** | **Location** | **Sequence** |
| *hBDNF_IXbAS* | exon IX | GTCCTCATCCAACAGCTCTTCTATC |
| *haBDNF_10AS* | exon 10 | CAAACCAATGGAACCAAGAAAGTC |
| *haBDNF_9AS* | exon 9 | GAGAAATAGGAGGTGAGCAGTC |
| *Dup_AS* | exon IX | CGTGTACAAGTCTGCGTCCTTATTG |
| *BDNFcod_AS* | exon IX | GACCCTCATGGACATGTTTGCAGC |
| *RACEhBDNF_IAS* | exon I | AACTCTCAACCACCTTGGCGACTA |
| *RACEhBDNF_IASn* | exon I | GACAAAGCAACTGGCATCGATGTCG |
| *RACEhBDNF_IIAS* | exon II | AACTGGGGCTCGCTTTCCAAACGCTC |
| *RACEhBDNF_IIASn* | exon II | CTCTCCAGCCCCGATCTCAGTGTGA |
| *RACEhBDNF_IIIAS* | exon III | TGAGGCATCCGGCCCGGCTGGGGA |
| *RACEhBDNF_IIIASn* | exon III | GGTCTCGGCAGCTCCCCTTCCCTT |
| *RACEhBDNF_IVAS* | exon IV | GTCAAAGTAACCATCAAGGCAGCTGC |
| *RACEhBDNF_IVASn* | exon IV | GAAAGACTTCGGCCCCAAAACTCCC |
| *RACEhBDNF_VAS* | exon V | GCGAACGCGAGCACACAATGAAATC |
| *RACEhBDNF_VhAS* | exon Vh | TCCCGCGCCCTCTGCAGAAAC |
| *RACEhBDNF_VhASn* | exon Vh | GGGAGGGAGCGAGTGAGAATC |
| *RACEhBDNF_VIAS* | exon VI | CAGCAGCGGTGGGTGTCTCATTAA |
| *RACEhBDNF_VIASn* | exon VI | AGCAGGAGGTGGAGGGGCGCA |
| *RACEhBDNF_VIIAS* | exon VII | CCTACCGCTGGGAACTGAAAGGGTCTG |
| *RACEhBDNF_IXAS* | exon IX | TTAGATGGCTTGTAAGCAAGTGCAA |
| *RACEhBDNF_IXASn* | exon IX | CCTTCTTCCCACTTTAGCAGC |
| *RACEhaBDNF_1AS* | exon 1 | CTGATGGCCGCGGCGACACACTC |
| *Prom_IAS* | exon I | CCTTGGCGACTACAGAAGACAAAGC |
| *Prom_IIAS* | exon II | AGTTAACCCAGTATACCAACCCGG |
| *Prom_IIIAS* | exon III | AACCCTCTAAGCCAGCGCCCGAAAC |
| *Prom_IVAS* | exon IV | GTCAAAGTAACCATCAAGGCAGCTGC |
| *Prom_VIAS* | exon VI | GGAGTCACATCGTGGTTCCGATTCTG |
| *Prom_VIIAS* | exon VII | ATTCCGCCTCCCAAGTTTTCCAAGCT |
| *Prom_1AS* | exon 1 | ACACTCGGCCACCTTCCTGATCTCGC |
