# Supplementary material for: Dissecting the human BDNF locus: Bidirectional transcription, complex splicing, and multiple promoters
Source: Genomics. 2007 Sep;90(3):397–406. doi: 10.1016/j.ygeno.2007.05.004 (PMC2568880; doi:10.1016/j.ygeno.2007.05.004)
Supplement: Supplementary Table 2 [file mmc2.doc]

**Supplementary Table 2.1 Exon-intron boundaries of human *BDNF*** gene

| Splice acceptor | Exon | Size | Splice donor | Intron | Size |
| --- | --- | --- | --- | --- | --- |
|  | I | 647 bp | GCACAATG**gt**gagtagc | 1 | 632 bp |
|  | IIa | 136 bp | ACCCCCAG**gt**agtcttc | 2a | 897 bp |
|  | IIb | 351 bp | AGCTCCGG**gt**tggtata | 2b | 682 bp |
|  | IIc | 434 bp | CAAAGAAG**gt**aagcacc | 2c | 599 bp |
|  | III | 237 bp | GAGCCCAG**gt**ccgagtc | 3 | ~ 18 kb |
|  | IV | 337 bp | CTGAAAAG**gt**gggtttg | 4 | 244 bp |
|  | Va | 72 bp | GCAGGAAG**gt**gcgggaa | 5a | 80 bp |
|  | Vb | 82 bp | GCGGGAAG**gt**gtgtctg | 5b | 70 bp |
|  | Vh | 225 bp | GGGAGGAG**gt**gaggaca | 5h | 187 bp |
|  | VIa | 306 bp | CCCGTGAG**gt**ttgtgtg | 6a | 515 bp |
|  | VIb | 324 bp | ACCCCGAG**gt**aggcaag | 6b | 497 bp |
|  | VII | 184 bp | CCCTCCAG**gg**aagttaa | 7 | ~ 25 kb |
| gctttac**ag**TTAAGTAA | VIII | 283 bp | TCATAAAG**gt**gagtaac | 8 | ~ 14 kb |
| tgtgttt**ag**GGATGGTA | VIIIh | 117 bp | GACTGGAG**gt**aggttgc | 8h | 478/1216 bp^a^ |
|  | IXabcd | ~ 4,8 kb^b^ |  |  |  |
| tcgtgac**ag**CATGAGCA | IXb | 107 bp | AATCTCAG**gt**tatatgc | 9b | 234 bp |
| tccctac**ag**TTCCACCA | IXd | ~ 3,7 kb |  |  |  |

^a^ The size of *BDNF* intron 8h is 478 bp when the transcription start site of exon IXabcd is considered to be the end of intron 8h and 1216 bp when the splicing acceptor site of exon IXb is considered to be the end of intron 8h.

^b^The size of *BDNF* exon IXabcd is ~ 4,8 kb when no splicing occurs subsequent to transcription starting from exon IXa

**Supplementary Table 2.2 Exon-intron boundaries of human *antiBDNF*** gene

| Splice acceptor | Exon | Size | Splice donor | Intron | Size |
| --- | --- | --- | --- | --- | --- |
|  | 1 | 47 bp | CCATCAGG**gt**aagggtac | 1 | ~ 33 kb |
| tttgctc**ag**CACTTCTC | 2 | 97 bp | GCAGAACT**gt**gagtcaa | 2 | ~ 44 kb |
| tccttgc**ag**GGGGTTAT | 3 | 69 bp | GCTTTTAG**gt**aagtacc | 3 | ~ 55 kb |
| tctttcc**ag**GGCTAGAG | 4 | 161 bp | CTAACCAG**gt**gagtttc | 4 | ~ 18 kb |
| ctccagc**ag**AAAGAGAA | 5a | 222 bp | ACACCTGG**gt**aggccaa | 5a | ~ 0,7 kb |
| ctccagc**ag**AAAGAGAA | 5b | 304 bp | TAACCATA**gt**aaggaa | 5b | ~ 0,6 kb |
| ctccagc**ag**AAAGAGAA | 5c | 380 bp | GAAAACTG**gt**tagggct | 5c | ~ 0,5 kb |
| ttcccac**ag**AGATACTC | 6 | 58 bp | TTTTACAG**gt**aaggaaa | 6 | ~ 6 kb |
| tccatga**ag**TTTCAGTG | 7 | 132 bp | CAACTGAG**gt**accgggt | 7 | ~ 10 kb |
| tgtttcc**ag**TATTGAAT | 8a | 844 bp | CTTCACAG**gt**attatta | 8a | ~ 0,8 kb |
| tcttttt**ag**CACCCAAC | 8b | 564 bp | CTTCACAG**gt**attatta | 8b | ~ 0,8 kb |
| ctgctac**ag**GAAATTCT | 9a | 158 bp | CGGATGGG**gt**aaaagac | 9a | ~ 20 kb |
| ctgctac**ag**GAAATTCT | 9b | 820 bp |  | 9b |  |
| tttttac**ag**GATTTTCC | 10 | 692 bp |  | 10 |  |
