# Supplementary material for: Dissecting the human BDNF locus: Bidirectional transcription, complex splicing, and multiple promoters
Source: Genomics. 2007 Sep;90(3):397–406. doi: 10.1016/j.ygeno.2007.05.004 (PMC2568880; doi:10.1016/j.ygeno.2007.05.004)
Supplement: Supplementary Legend [file mmc3.doc]

**Supplementary Fig. 1**. Alignment of the nucleotide sequences of human, rat and mouse *BDNF* genes. The nucleotide sequences of the genomic regions of the *BDNF* genes spanning exons I – III, IV – VII, VIII and VIIIh – IX were aligned. Asterisks indicate identical nucleotides in the human, rat and mouse genes. Sequences in *bold* represent the exons of the human *BDNF* gene. *Underlined* sequences of the human *BDNF* gene represent the promoter regions cloned for CAT assay analyses of promoter activity in this study. *Double-underlined* sequences of the human *BDNF* gene represent the coinciding promoter regions of adjacent promoter regions analysed in this study. *Filled triangles* point to the transcription start sites of the human *BDNF* determined by 5’ RACE in this study. *Open circles* indicate transcription starts sites determined for rat *BDNF* [12,15,17]. *Vertical lines* on the right of the alignment mark the exon regions of the *BDNF* genes. *Double vertical lines* on the right of the alignment mark the protein coding regions of the *BDNF* exons. *Arrows* above ATG nucleotide triplets in exons I, VII, VIII and IX indicate the putative translation start sites of the human *BDNF*. *Horizontal line ending with a filled bulb* above TAG nucleotide triplet in exon IX indicates the translation stop codon of the human *BDNF*. *Square brackets opening to the left* and *to the right* indicate the splicing donor and splicing acceptor sites, respectively.

**Supplementary Fig. 2**. Complementary region of human *BDNF* and *antiBDNF* transcripts. *BDNF* is the upper sequence, *antiBDNF* is the lower sequence. The protein coding sequence and its 5’ adjacent sequence of *BDNF* exon IXd are shown. The complementary region is in bold and the sequences of both *BDNF* exon IXd and *antiBDNF* exon 5 are in *uppercase letters*. *Square brackets opening to the left* and *to the right* indicate the splicing donor and splicing acceptor sites, respectively for the *BDNF* strand. *Square brackets opening to the left* and *to the right* indicate the splicing acceptor and splicing donor sites, respectively for the *antiBDNF* strand.
