# Supplementary material for: Dissecting the human BDNF locus: Bidirectional transcription, complex splicing, and multiple promoters
Source: Genomics. 2007 Sep;90(3):397–406. doi: 10.1016/j.ygeno.2007.05.004 (PMC2568880; doi:10.1016/j.ygeno.2007.05.004)
Supplement: Supplementary Figure 1 [file mmc4.pdf]

Alignment of human, rat and mouse *BDNF* genes

```
human_BDNF      ------CAACCTGCCGCCTCCCCTCCCGTCCCTGCCT-TGGCCCGCGGCTCCGCTCTCCG 53
rat_BDNF        ---------ACTAGAGCGGATCCTCCCCTCCTAGCCTATACCTCTTCGCTCAGAGATCTA 51
mouse_BDNF      AGCTGGGAGACTAGCGCCGATCTTCCCCTCCTAGCCTACACCTTTTCGCTCAGAGCTC-- 58
                          **   **    * **** ***  ****    *     **** *   **

human_BDNF      CGGCTCGCCTTCCG-ACTTAGCTGCGCACCGGGGCTGTTAACTCACATTTGGGAAGCCAT 112
rat_BDNF        GGGCTCGCAGGGCGGACCTAGCTGCACACAGAGGCTGTTAACTCTCATTTGAGAAGCCAT 111
mouse_BDNF      -------CAGGGCGGACCTAGCCGCGCACAGAGGCTGTTAACTCTCATTTGAGAAGCCAT 111
                       *    ** ** **** ** *** * ************ ****** ********

human_BDNF      AACCCATTAGAGCAAACGCAGTCATAACTTCATTCAACTCA-GCCGCTCGAGAGCTCGGC 171
rat_BDNF        AAGCCATTAGAGCAAACGCAGTCATAACTTCATTCAACTCAAGCCGCTTGAGAGCTTGGC 171
mouse_BDNF      AAGCCATTAGAGCAAACGCAGTCATAACTTCATTCAACTCAAGCCGCTTGAGAGCTTGGC 171
                ** ************************************** ****** ******* ***

human_BDNF      TTACACAGGTTCCTGTGGGCAACTAGTGGCTCGCCTTGGTGCCTCTCGCCTAGTCATCAG 231
rat_BDNF        TTACACCGGTTCCTGTGGGCAACTAGTGGCTCGCCCGGGTGCCTCTCGCCTAGTCATCAG 231
mouse_BDNF      TTGCACAGGTTCCTGTGGGCAACTAGTGGCTCGCCCGGGTGCCTCTCGCCTAGTCATCAG 231
                ** *** ****************************  ***********************

human_BDNF      TACCTAAGAGGAAAAGGGAAAGTTGTTGGGCTGGTTCGCGCTTCGACGCATGCAGATGTT 291
rat_BDNF        TCCCTAAGAGGAAAAGGGAAAGTTGTGGGGCTGATGCGCTCTTCGATTCACGCAGTTGTT 291
mouse_BDNF      TCCCTAAGAGGAAAAGGGAAAGTTGTGGGGCTGATGCGCTCTTCGATTCACGCAGTTGTT 291
                * ************************ ****** * *** ******  ** **** ****

human_BDNF      CCCAAGGACAAGTCACTTACTCGCCCCCC--------TCCCCCCAGTCCCCATTTGATCA 343
rat_BDNF        CCCTAGAACAAGTCACTCCTGCTCCATCA---------CCCCTCCCCCCCCATTTGATCA 342
mouse_BDNF      CCCTAGAACAAGTCACTCCCGCTCCATCACCCAGCCCCCCCCCCCCCTCCCATTTGATCA 351
                *** ** **********    * **  *          **** *    ************

human_BDNF      TCACTCACGACCTCATCGGCTGGAGACCCTTAGTCATGATGGGGGAGGGGGAGGGGCACG 403
rat_BDNF        TCACTCACGACCACGTCCGCTGGAGACCCTTAGTCATGGTGGGGGAGGGG------CACG 396
mouse_BDNF      TCACTCACGACCACGTCCGCTGGAGACCCTTAGTCATGGTGGGGGAGGGG------CACG 405
                ************ * ** ******************** ***********      ****

human_BDNF      AACTTTTCTAAGAAGTTTCCTTTTTTTACCCAGAGAGTCACAGTGAGTCGGTCACGTAAA 463
rat_BDNF        AACTTTTCTAAGAAGTTTCCTTTTT--ACCAAGGGAGTCACAGTGAGTTGGTCACGTAAC 454
mouse_BDNF      AACTTTTCTAAGAAGTTTCCTTTTT--ACCAAGGGAGTCACAGTGAGTTGGTCACGTAAC 463
                *************************  *** ** ************** **********

human_BDNF      CAGCG-AGGTTAGTCGTCGCCGTTGCCGCCCCCCACCCCCTCCCT-------------GC 509
rat_BDNF        TGGCTCAGAGAGGCTGCCCTGGCCCCCTCCCCTCGCCCCCTCCCCTCGCCCCCTCCCCGC 514
mouse_BDNF      TGGCTCAGAGAGGCTGCCCTGGCCCCCTCCCCTCGCCCCCTCCCC-------------GC 510
                  **  **    *  * *   *   ** **** * *********              **
                                 ▼    ○                     ○○○
human_BDNF      TGCGCTTTTCTGGTATTATTATTAAAGCGGTAGTCTGCCGGCGCTGATAAGCAACAAGTT 569
rat_BDNF        TGCGCTTTTCTGGTATTCTTATTAAAGCGGTAGCCGGCTGGTGCAGGAAAGCAACAAGTT 574
mouse_BDNF      TGCGCTTTTCTGGTATTCTTATTAAAGCAGTAGCCGGCTGGTGCAGAAAAGCAACAAGTT 570
                ***************** ********** **** * ** ** ** *  ************

human_BDNF      CCCCAGCGGTCTTCCCGCCCTAGCCTGACAAGGCGAAGGTTTTCTTACCTGGCGACAGGG 629
rat_BDNF        CCCCAGCGGTCTTCCCGCCCTAGCCTGACAAGGCGAAGGTTTTCTTACCTGGCGACAGGG 634
mouse_BDNF      CCCCAGCGGTCTTCCCGCCCTAGCTTGACAAGGCGAAGGGTTTCTTACCTGGCGACAGGG 630
                ************************ ************** ********************

human_BDNF      AAATCTCCCGAGCCGAATTCAGCTTCGCCGGAGCCCCAGGTGTGACCTGCGTAGTGGGCA 689
rat_BDNF        AAATCTCCTGAGCCGAGCTCATCTTTGCCACAGCCCCAGGTGTGACCTGAGCAGTGGGCA 694
mouse_BDNF      AAATCTCCTGAGCCGAGCTCATCTTTGCCAGAGCCCCAGGTGTGACCTGAGCAGTGGGCA 690
                ******** *******  *** *** ***  ****************** * ********
                                                                         ▼
human_BDNF      AGGGAGCGGTGTGCAGGCTGAGTTTTTTTTTTTACAGGGGTACCCTGAAACTCC-TCACT 748
rat_BDNF        AAGGAGCGGCGTGCAAATTGGATTATTTGT---ATGGGGGTACTCTGAAACTCCCTCACT 751
mouse_BDNF      AAGGATCGGCGTGCAAATTGGATTATTTTT---ATGGGGGTACTCTGAAACTCCCTCACT 747
                * *** *** *****   **  ** *** *   *  ******* ********** *****

human_BDNF      TTCTCTGGGAACTTTCAGTGCCAGGACCCAGTAACGGGCGGTTAGAAGGCAGCCCTAGGA 808
rat_BDNF        TTTTCTGGGAACTTTTTGTGCTAGGGCGCAGTGACAGGCGTTGAGAAAGCTGCTTCAGGA 811
mouse_BDNF      TTCTCTGGGAACTTTTTGTGCTAGGGCTCAGTGACAGGCGTTGAGAAAGCTGCTTCAGGA 807
```

```
                 ** ************  **** *** * **** ** **** * **** ** **   ****

human_BDNF       AACACCTGCTACATAGCAGGGCAGTTGGGCAATCATTGGTAACCTCGCTCATTCATTAGA 868
rat_BDNF         AACGCCCGCTATATAGCAGGGCAGTTGGACAGTCATTGGTAACCTCGCTCATTCATTAGA 871
mouse_BDNF       AACGCCCGCTATATAGCAGGGCAATTGGACAGTCATTGGTAACCTCGCTCATTCATTAGA 867
                 *** ** **** *********** **** ** ****************************

human_BDNF       ATCACGTAAGAACTCAAAAGGAAACGTGTCTCTCGGAGTGAGGGCGTTTGCGTAAATCTA 928
rat_BDNF         ATCACGTAAGAACTCAAAGGGAAACGTGTCTCTCAGAATGAGGGCGTTTGCGTAAATCTA 931
mouse_BDNF       ATCACGTAAGAACTCAAAGGGAAACGTGTCTCTCAGAATGAGGGCGTTTGCGTAAATCTA 927
                 ****************** *************** ** **********************

human_BDNF       TAGGTTTTTCGACATCGATGCCAGTTGCTTTGTCTTCTGTAGTCGCCAAGGTGGTTGAGA 988
rat_BDNF         TAGGTTTTTCAACATCGATGCCAGTTGCTTTGTCTTCTGTAATCGCCAAGGTGGATGAGA 991
mouse_BDNF       TAGGTTTTTCAACATCGATGCCAGTTGCTTTGTCTTCTGTAGTCGCCAAGGTGGATGAGA 987
                 ********** ****************************** ************ *****

human_BDNF       GTTTAAGCTT-GCGGATATTGCAAAGGGTTATTAGATTCATAAGTCACACCAAGTGGTGG 1047
rat_BDNF         GTTGAAGCTT-GCGGATATTGCAAAGGGTTATTAGATTCATAAGTCACACCAAGTGGTGG 1050
mouse_BDNF       GTTGAAGCTTTGCGGATATTGCGAAGGGTTATTAGATTCATAAGTCACACCAAGTGGTGG 1047
                 *** ****** *********** *************************************

human_BDNF       GCGATCCACTGAGCAAAGCCGAACTTCTCACATGATGACTTCAAACAAGACACATTACCT 1107
rat_BDNF         GCGATCCACTGAGCAAAGCCGAACTTCTCACATGATGACTTCAAACAAGACACATTACCT 1110
mouse_BDNF       GCGATCCACTGAGCAAAGCCGAACTTCTCACATGATGACTTCAAACAAGACACATTACCT 1107
                 ************************************************************

human_BDNF       TCCAGCATCTGTTGGGGAGACGAGATTTTAAGACACTTGAGTCTCCAGGACAGCAAAGGC 1167
rat_BDNF         TCCAGCATCTGTTGGGGAGACGAGATTTTAAGACACT-GAGTCTCCAGGACAGCAAAGCC 1169
mouse_BDNF       TCCTGCATCTGTTGGGGAGACAAGATTTTAAGACACT-GAGTCTCCAGGACAGCAAAGCC 1166
                 *** ***************** *************** ******************** *

human_BDNF       ACAATGGTGAGTAGCAATAAAACCTGCATTATAATTGAAAAA---TCTTGACATGTTGCT 1224
rat_BDNF         ACAATGGTGAGTAGCAATAAACCCTGCATTATAATTGAAAAAAAATCTTGACATGTTGCC 1229
mouse_BDNF       ACAATGGTGAGTAGCAATAAACCGTGCATTATAATTGAAAAA---TCTTGACATGTTGCC 1223
                 ********************* * ******************   **************

human_BDNF       TAACAACGGGCATATCACGGCTCTTCCTAGCACTTCACACGCCAAAGAACAGCAGCTACT 1284
rat_BDNF         TAACAACAGGCATATCACGGCTCTTCCTAGCACTTCACACGCCAAAGAACAGCAGCTATT 1289
mouse_BDNF       TAACAACAGGCATATCATGGCTCTTCCTAGCACTTCACACGCCAAAGAACAGCAGCTATT 1283
                 ******* ********* **************************************** *

human_BDNF       CAGGCCAGGGGAATCGGGTTTTTACACAGTGCAACTTTAATTGGAATCATTTGAGATTTG 1344
rat_BDNF         CAGGCCAGGG-AATCGGGTTT--ACCCACTGCAACTTTAATTGGAATCATTTGAGATTTA 1346
mouse_BDNF       CAGGCCAGGGGAATCGGGTTT--ACCCACTGCAAGTTTAATTGGAATCATTTGAGATTTA 1341
                 ********** **********  ** ** ***** ************************

human_BDNF       ACACAGCTATGTGGAACTGCGTGGAACAAACTTGGAGCTGGGTGGGGGGGTGTGTGTTAT 1404
rat_BDNF         ATACAGCAGCATGATACTGCGTGGAACAAACTTGGGACTGGA---GGGGGT---TGTTAT 1400
mouse_BDNF       ACACAGCAGTGGGGAACTGCGTGGAACAAACTTGGAACTGGA---GGGGGT---TGTTAT 1395
                 * *****     *  ********************  ****    ******   ******

human_BDNF       ATTGGTTGTTCAAGGCTGATGCTTGTCTCTCAGCAGTCTTGCATTCTATTCTTTTCCTTA 1464
rat_BDNF         ACTGGCTTTTCAAAGCTGATGCTTGTCTGACAACCGTCTTGTATTGCATTCTTTGCTTTA 1460
mouse_BDNF       ACTGGCTGTTCAAAGCTGATGCTTGTCTGACAACCGTCTTGTATTCCATCCTTTGCTTTA 1455
                 * *** * ***** **************  ** * ****** ***  ** **** * ***

human_BDNF       ATGTGTATGGTGTATGATCATATTCTATGATTTATATGTGGGCATGTAATTGACATTTGC 1524
rat_BDNF         CTGT--ATGGTGTGTGAGCATATTGTATGATTTATATGTGGGCATATAATTGACATCCGC 1518
mouse_BDNF       CTGT--ATGGTGTGTGAGCATATTGTATGATTTATATGTGGGCATATAATTGACATCCGC 1513
                  ***  ******* *** ****** ******************** **********  **

human_BDNF       AAGGGGGTTAATTTCCATCTAAAAACAATAATGCTGTTAGAGGTTGGGGTTAGGGGGTGG 1584
rat_BDNF         AAGGGGGTTAATTTCAATCTAAAAACATTAATGTTCTTAGAGTTTGGGGCTAGGGGGTGG 1578
mouse_BDNF       AAGGGGGTTAATTTCAATCTAAAAACAATAATGTTATTAG--TTTGGGGCT--GGGGTGG 1569
                 *************** ************ **** * ****   ****** *  *******

human_BDNF       AGTGGGGGTAAGGGTGGGGTAAACACTGGGAGTTTAGGTGTAGATGGGGGGTGGGGTTGG 1644
```

EXON I

```
rat_BDNF       AG--AGGGTAAGAGTGGAGTAGAGACTAAGAGTTTAGGTGAGGATAGGGGTGGAGTTGGG 1636
mouse_BDNF     AG--AGGGTAAGAGTGGGTTAGAGACTAAGAGTTTAGGTGAGGATAGTGGTGGAGTTGGG 1627
               **   ******* ****  ** * ***  ***********  *** * **  * * * **

human_BDNF     GGGGAGAGAAATAAGTCAGAAGTGCATATCACCGGT--AATGGGTAATCCTCTCGTAGAA 1702
rat_BDNF       GGAGAGAGGAATAAGTCAGAAGTGCCTATCTATAATGAAATGGGTAATGCTCTCATACAT 1696
mouse_BDNF     GGCGAGAGGAATAAGTCAGAAGTGTCTATCTATAATGAAATGGGTAATGCTCGCATACGT 1687
               ** ***** ***************  ****      *  ********** *** * **

human_BDNF     GAAAAGGTTCTCATCAACATGTGATCAACTATTAACAGGATGGCTTTGGCAAAGCCATCC 1762
rat_BDNF       GGAAAGGGTCTCATCAACATGTGATCAACTATTAACAGGATGGCTTTGGCAAAGCCATCC 1756
mouse_BDNF     GGAAAGGGTCTCATCAACATGTGATCAACTATTAACAGGATGGCTTTGGCAAAGCCATCC 1747
               * ***** ****************************************************

human_BDNF     GCACGTGACAAACCGTAAGGAAGTGGAAGAAACCGTCTAGAGCAATATCAAGTATCACTT 1822
rat_BDNF       GCACGTGACAAAACGTAAGGAAGTGGAAGAAACCGTCTAGAGCAATATCAAGTACCACTT 1816
mouse_BDNF     ACACGTGACAAAACGTAAGGAAGTGGAAGAAACCGTCTAGAGCAATATCAAGTACCACTT 1807
                *********** ******************************************* *****

human_BDNF     AATTAGAGA----TTTTTAAGCCTTTTCCTCCTGCTGTGCCGGGTGTGTAATCCGGGCGA 1878
rat_BDNF       AATTAGAGAATATTTTTTTAACCTTTTCCTCCTGCTGCGCCGGGTGTGTGATCCGGGCGA 1876
mouse_BDNF     AATTAGAGAATATTTTTTTAACCTTTTCCTCCTCCTGCGCCGGGTGTGTGATCCCGGAGA 1867
               *********    ***** * ************ *** *********** **** ** **

human_BDNF     TAGGAGTCCATTCAGCACCTTGGACAGAGCCAACGGATTTGTCCGAGGTGGCGGTACCCC 1938
rat_BDNF       GCAGAGTCCATTCAGCACCTTGGACAGAGCCAGCGGATTTGTCCGAGGTGGTAGTACTTC 1936
mouse_BDNF     GCAGAGTCCATTCAGCACCTTGGACAGAGCCAGCGGATTTGTCCGAGGTGGTAGTACTTC 1927
                  **************************** ******************   ****  *

human_BDNF     CAGGTAGTCTTCTTGGCCCCGCTGTAAAGCCAACCCTGTGTCGCCCCTTAAAAAGCGTCTT 1998
rat_BDNF       ATCCAGGTATTCTTTTCCTCGCTGTCAAGCCAACCCGGTGTCGCCCCTTAAAAAGCGTCTT 1996
mouse_BDNF     ATCCAGGTATTCTTTTCCTCGCTGTCAAGCCAACCCGGTGTCGCCCCTTAAAAAGCGTCTT 1987
                     ** *****  ** ****** ********** ***********************

human_BDNF     TTCTGAGGTTCGGCTCACACTGAGATCGGGGCTGGAGAGAGAGTCAGATTTTGGAGCGGA 2058
rat_BDNF       TTCCGAGGTTCGGCTCACACTGAGATCGGGGCTGGAGAGAGAGTCAGATTTTGGAGCGGA 2056
mouse_BDNF     TTCCGAGGTTCGGCTCACACCGAGATCGGGGCTGGAGAGAGAGTCAGATTTTGGAGCGGA 2047
               *** **************** ***************************************

human_BDNF     GCGTTTGGAAAGCGAGCCCCAGTTTGGTCCCCTCATTGAGCTCGCTGAAGTTGGCTTCCT 2118
rat_BDNF       GCGTTTGGAGAGCCAGCCCCAGTTTGGTCCCCTCATTGAGCTCGCTGAAGTTGGCTTCCT 2116
mouse_BDNF     GCGTTTGGAGAGCGAGCCCCAGTTTGGTCCCCTCATTGAGCTCGCTGAAGTTGGCTTCCT 2107
               ********* *** **********************************************

human_BDNF     AGCGGTGTAGGCTGGAATAGACTCTTGGCAAGCTCCGGGTTGGTATACTGGGTTAACTTT 2178
rat_BDNF       AGCGGTGTAGGCTGGAATAGACTCTTGGCAAGCTCCGGGTTGGTATACTGGGTTAACTTT 2176
mouse_BDNF     AGCGGTGTAGGCTGGAATAGACTCTTGGCAAGCTCCGGGTTGGTATACTGGGTTAACTTT 2167
               ************************************************************

human_BDNF     GGGAAATGCAAGTGTTTATCTCCAGGATCTAGCCACCGGGGTGGTGTAAGCCGCAAAGAA 2238
rat_BDNF       GGGAAATGCAAGTGTTTATCTCCAGGATCTAGCCACCGGGGTGGTGTAAGCCGCAAAGAA 2236
mouse_BDNF     GGGAAATGCAAGTGTTTATCACCAGGATCTAGCCACCGGGGTGGTGTAAGCCGCAAAGAA 2227
               ******************** ***************************************

human_BDNF     GGTAAGCACCAGGGCGGGGACCCCTTGCATCCCCAATTCTTGAGCTATTTTGATACT--G 2296
rat_BDNF       GGTAAGCACCGGGGCGGGGACCC--TGCATCCCCAATTCTTGAGCTATTTTGATACTCGA 2294
mouse_BDNF     GGTAAGCACCGGGGCGGGGACCC--TGCATCCCCAATTCTTGAGCTATTTTGATACTCGA 2285
               ********** ***********   ********************************

human_BDNF     TCTTCCGGAGAGGACGCGTGGTGGAGGGGAGGAGGTAGAGGGAGAGCATGAGAGGGGGTT 2356
rat_BDNF       TCTTCCGGAAAGGACGCGTAGTGGAGCGGAGGAAGTGGAGGGAGAGCCTGAGAAATGGTT 2354
mouse_BDNF     TCTTCCGGAAAGGACGCGTAGTGGAGAGGAGGAAGTGGAGAGAGAGCATGAGAAATGGTT 2345
               ********* ********* ****** ****** ** *** ****** *****    ****

human_BDNF     GTTTCTTGGTATTTGCCCAGTTTGAATTGCCCTAGGTGAGAACCCTGGGGCAAAGGGAGA 2416
rat_BDNF       GTTTCTTGGTATTTGTCTAGTTTAAATTGACCTAGGTGAGAACC-TGGGGCAAATCGAGG 2413
mouse_BDNF     GTTTCTTGGTATTTGGCTAGTTTAAATTGACCTAGGTGAGAACC-TGGGGCAAATCGAGG 2404
               *************** * ***** ***** ************** *********  ***
```

EXON II

```
human_BDNF      AAGAAAAAAAAGAAACTCAGTCTTCCTGCGGATATAATGAGTTTAGTTAACTTGGACCTG 2476
rat_BDNF        TTGGG------GAAGCTCAAGCTTCCTGCGGATATAATGAGTTTAGTTAACTTGGAACAG 2467
mouse_BDNF      TGGAG------GAAGCTCAATCTTCCTGCGGATATAATGAGTTTAGTTAACTTGGAACTG 2458
                  *        *** ****  *********************************** * *

human_BDNF      CAAATGTCTGATTCAAATGTAAGATTTATCTCTCTTTTTCTCCTCTTCACCTCCCTCTTT 2536
rat_BDNF        CAAATATCTGATTCAAATGTAAGATTT----CTCTCATTTCCCCCTTTCCCTCCCTCTTT 2523
mouse_BDNF      CAAATATCTGATTCAAATGTAAGATTT----CTCTCATT-CCCCCTTTCCCTCCCTCTTT 2513
                ***** *********************    ****  **  ** ***  ***********

human_BDNF      TCCGTTCTCTTTGCTGGTGTGT---GTGTGTGTGTGTACAGTAGATTCATTACTAATTAT 2593
rat_BDNF        TCCGTTCTTTTTGCTTGTGTGTGGTGTGTGTGCGTGTACAGTAGATTCATTACTAATTAT 2583
mouse_BDNF      TCCGTTCTTTTTGCTTGTGTGTGGTGTGTGTGCGTGTACAGTAGATTCATTACTAATTAT 2573
                ******** ****** ******   ******* ***************************

human_BDNF      GAAGCTTTT-GCAAAACATTCGAATTCCTAAAATTTGACTTTGTAGCATTTAGAATCAGG 2652
rat_BDNF        GAAGCTTTTTACACAACATTCGATTTCCTAAGATTTGACTTTGTACCATTTAGAATCAGA 2643
mouse_BDNF      GAAGCTTTTTACACAACATTCGATTTCCTAAGATTTGACTTTGTACCATTTAGAACAAGA 2633
                *********  ** ********* ******* ************* *********  **

human_BDNF      CGGTGGAGGTGGTGTGCGGTGGGGA--GAGGAGGTGGAGGTTGGGAAGAGGGAAGGAGGT 2710
rat_BDNF        GGATGGAGGGAGTGTGCAATGGGGAAAGGGGAGGTGGAGGCTGGGA-GAGGGAAGGAGGT 2702
mouse_BDNF      GGATGGAGGGAATGTGCAGTGGGGAAAGAGGAGGTGGAAGCTGGGA-GAGGGACGGAGGT 2692
                 * ******   *****  ******  * ********* * ***** ****** ******

human_BDNF      AAAGCTAAACCTCCAACACAAAAAAATGAATCAAGGTAATTTCAGCTCTTCTAGTGAGAA 2770
rat_BDNF        TA-GCTAAATATCCAC-----AAAAATGAATCAAGGTAATTTCAGCTCCTCTAGTGAAGA 2756
mouse_BDNF      TA-GCTAAATACCCAC-----AAAAATGAATCAAGGTAATTTCAGCTCCTCTAGTGAGGA 2746
                 * ******   ***      *************************** ********  *

human_BDNF      GGATTCATTCTCTCTGTATCCCTCCCTCCCTCTCTTTCCCCC-TCCCTCCCTCCTTCCCG 2829
rat_BDNF        GGATTCATTCTTTCGGTATCCCTGCCTCCCTCTCTTTCCCCC-TCCCTCCCTCCTTCCCG 2815
mouse_BDNF      GGATTCATTCTCTCGGTATCCCTGCCTCCCTCTCTTTCCCCCCTCCCTACCTCCTTCCCG 2806
                *********** ** ******** ****************** ***** ***********
                        ▼                                             ▼    ○
human_BDNF      CCCCCCTTCTTCCACCCCGCCCCCTCCTCCAGCCTCCATCCCTCCCTCATTCTATCTCTT 2889
rat_BDNF        CCCCCCTTCTTCTACCCCGCCCCCTCCTCCTGCCTCCATCCCTCCCTCATTTTCTCTCTA 2875
mouse_BDNF      CCCCCCTT-TTCCACCCCGCCCCCTCCTCCTGCTTCCATCCCTCCCTCATTTTCTCTCTA 2865
                ******** *** ***************** ** ***************** * *****

human_BDNF      CCTCTCCGTCGCCCTCGC--TCCTCGCTGGATGCTTCTTTCT-----GGGTTTTCTTTTT 2942
rat_BDNF        CCTCTCCATCGCCCTCACGATTCTCGCTGGATAGTTCTTTATGTTTTGGGTGATTTTTGT 2935
mouse_BDNF      CCTCTCCATCGCCCTCACGATCCTCGATGGATAGTTCTTTATGTTT-GGGTGATTTTTTT 2924
                ******* ******** *  * **** *****  ****** *     ****  * *** *
                              ○
human_BDNF      TTTTT----------------------------------CCCTTCTGTCCTCCCTCCCCG 2968
rat_BDNF        TTTTTGTTTGTTTGGTTTTTTTTTTTTTTAATTTTTACCCCTTTCTATTTTCCCTCCCCG 2995
mouse_BDNF      TTTTT-------------------------------ACCCCTTTCTATCATCCCTCCCCG 2953
                *****                                  ** **** *  **********

human_BDNF      CGAGTTTCGGGCGCTGGCTTAGAGGGTTCCCGCTTTCTCAAGGGAAGGGGAGCTGCCGAG 3028
rat_BDNF        AGAGTTCCGGGCTCTGGCTTGGAGGGCTCCTGCTTTCTCAAGGGAAGGGGAGCCGCTGAG 3055
mouse_BDNF      AGAGTTCCGGGTGCTGGCTTGGAGGGCTCCTGCTT-CTCAAGGGAAGGGGGGCCGCTGAG 3012
                 ***** ****  ******* ***** *** **** ************** ** ** ***

human_BDNF      ACCGCGCTCCGCTCCCCAGCCGGGCCGGATGCCTCACTGAGCCCAG]GTCCGAGTCAGTCG 3088
rat_BDNF        ACTGCGCTCCACTCCCT-GCCGGGCTGGATGCTTCATTGAGCCCAG]GTCCGAGTCAGGCG 3114
mouse_BDNF      ACTGCGCTCCACTCCCT-GCCGGGCCGGATGCTTCATTGAGCCCAG]GTCCGAGTCAGGCG 3071
                ** ******* *****  ******* ****** *** ******************** **

human_BDNF      GGGTAACTCAGGGAAAGGGGAGC----CTCCGCCTGGGAGTAGAAGGTCCTTTCCGGACC 3144
rat_BDNF        GGGTAACTCAGGGAAGGGGGGGGGAGGCTCCGCTCGGGAGTGGAAGGTCCTTTCTAGATC 3174
mouse_BDNF      GGGTAACTCAGGGAGGAGGGGGGT--GCTCCGCTCCGGAGTGGAAGGTCCTTTCTAGATC 3129
                **************   *** *     ******   ***** ************  ** *

human_BDNF      GAAGAG 3150
rat_BDNF        GAAGAG 3180
```

EXON III

```
mouse_BDNF      GAAGAG 3135
                ******
```

< ~ 17,5 kb >

```
human_BDNF      ---CCCCACCCTCGAATCACCTACCCCCACTCTGGTTAAAGCAGAAGACTTTTT------ 51
rat_BDNF        CAACCCCACCCACCCCTCACC---CTAAATTTTGGTTAAAGATTTAAAAAAAAA------ 51
mouse_BDNF      -------ACCCACCCCTCACC---CTAAATCCTGGTTAAGTATATATACATATATATATT 50
                       **** *   *****   *   *   *******      * *

human_BDNF      -ATTTATCTTGGCTGCCCTGGTTCGTTATTAAAAGGGTTAGCTTATACGTGTGTTTGCTG 110
rat_BDNF        -ATGTGTTGTTGCTGCC-TAGATGATGAAAGGTTTGGCTTCTGTGTGCGTGAGTTCGCTA 109
mouse_BDNF      GATGTGTTGTTGCTGCC-TAGATAATGACAGGCTTGGCTTCTGTGTGCGTGAATTTGCTA 109
                 ** * *  * ****** * * *  * *       ** *    * * ****  ** ***

human_BDNF      GGGCTGGAAGTGAAAACATCTGCAAAAGCATGCAATGCCCTGGAACGGAACTCTTCTAAT 170
rat_BDNF        GGACTGGAAGTGGAAACGTCTACAAA-GCATGCAATGCCCTGGAACGGAATTCTTCTAAT 168
mouse_BDNF      GGACTGGAAGTGAAAACATCTACAAA-GCATGCAATGCCCTGGAACGGAATTCTTCTAAT 168
                ** ********* **** *** **** *********************** *********

human_BDNF      AAAAGATGTATCATTTTAAATGCGCTGAATTTTGATTCTGGTAATTCGTGCACTAGAGTG 230
rat_BDNF        AAAAGATGTATCATTTTAAATGCGCGGAATTCTGATTCTGGTAATTCGTGCACTAGAGTG 228
mouse_BDNF      AAAAGATGTATCATTTTAAATGCGCGGAATTCTGATTCTGGTAATTCGTGCACTAGAGTG 228
                ************************* ***** ****************************

human_BDNF      TCTATTTCAGAGGCAGCGGAGGTATCATATGACAGCGCACGTCAAGGCACCGTGGAGCCC 290
rat_BDNF        TCTATTTC-GAGGCAGAGGAGGTATCATATGACAGCTCACGTCAAGGCAGCGTGGAGCCC 287
mouse_BDNF      TCTATTTC-GAGGCAGAGGAGGTATCATATGACAGCTCACGTCAAGGCAGCGTGGAGCCC 287
                ******** ******* ******************* ************ **********

                              ▼○  ▼○  ○   ○
human_BDNF      TCTCGTGGACTCCCACCCACTTTCCCATTCACCGCGGAGAGGGCTGCTCTCGCTGCCGCT 350
rat_BDNF        TCTCGTGGACTCCCACCCACTTTCCCATTCACCGAGGAGAGGACTGCTCGCGCTGCCGCT 347
mouse_BDNF      TCTCGTGGACTCCCACCCACTTTCCCATTCACCGAGGAGAGGACTGCTCTCGCTGCCGCT 347
                ********************************** ******* ****** **********

                                           ▼                   ○ ○ ○○○○ ○○○○
human_BDNF      C----------CCCCCGGCGAACTAGCATGAAATCTCCCTGCCTCTGCCGAGATCAAATG 400
rat_BDNF        CCCCCCACCCACCCCCGGCGAGCTAGCATGAAATCTCCCAGTCTCTGCCTAGATCAAATG 407
mouse_BDNF      CCCCCCACCCACCCCCGGCGAGCTAGCATGAAATCTCCCAGCCTCTGCCTAGATCAAATG 407
                *          ********** ***************** * ******* **********

                                          ▼
human_BDNF      GAGCTTCTCGCTGATGGGGTGCGAGTATTACCTCCGCCATGCAATTTCCACTATCAATAA 460
rat_BDNF        GAGCTTCTCACTGAAGGCGTGCGAGTATTACCTCCGCCATGCAATTTCCACTATCAATAA 467
mouse_BDNF      GAGCTTCTCGCTGAAGGCGTGCGAGTATTACCTCCGCCATGCAATTTCCACTATCAATAA 467
                ********* **** ** ******************************************

human_BDNF      TTTAACTTCTTTGCTGCAGAACAGAAGGAGTACATACCGGGCACCAAAGACTCGCGCCCC 520
rat_BDNF        TTTAACTTCTTTGCTGAAGAACAG---GAGTACATATCGGCCACCAAAGACTCGC-CCCC 523
mouse_BDNF      TTTAACTTCTTTGCTGCAGAACAG---GAGTACATATCGGCCACCAAAGACTCGC-CCCC 523
                **************** *******   ********* *** ************** ****

human_BDNF      CTCCCCCCTTTAATT-AAGCGAAGGGAACGTGAAAAAATAATAGAGTGTGGGAGTTTTGG 579
rat_BDNF        CTCCCCCTTTTAACTGAAGAGAAGGGGAAAT-ATATAGTAA--GAGTCTAGAACCTTGGG 580
mouse_BDNF      -TCCCCCTTTTAACTGAAGAGAAGGGGAAAT-ATATAGTAA--GAGTCTAGAACCTTGGG 579
                 ****** ***** * *** ****** *  * * * * ***  **** * * *  ** **

human_BDNF      GGCCGAAGTCTTTCCCGGAGCAGCTGCCTTGATGGTTACTTTGACAAGTAGTGACTGAAA 639
rat_BDNF        GACCG--GTCTTCCCCAGAGCAGCTGCCTTGATGTTTACTTTGACAAGTAGTGACTGAAA 638
mouse_BDNF      GACCG--GTCTTCCCCAGAGCAGCTGCCTTGATGTTTACTTTGACAAGTAGTGACTGAAA 637
                * ***  ***** *** ***************** *************************

human_BDNF      A-GGTGGGTTTGTTTTCTTTCTTTCTCTTT-CCGTTTTTTCTGT------TTGGTCGGCT 691
rat_BDNF        AAGGTGGGTTTCTTTTCTTTCTCTTTCCTCACCCCCCCGCCCCCC-----TTGGTCTGCT 693
mouse_BDNF      AAGGTGGGTTTCTTTTCTTTCTCTTTCCTCACCCCCCCCCCCGCCCCCGTTTGGTCTGCT 697
                * ********* ********** * ** *  **       *         ****** ***

human_BDNF      AGAAAGCGTGTGGCTTTAGCGAGGTCTGTCATTGCCTGGGCTTCCTGGCTGGAACAAGTA 751
rat_BDNF        GGGAAGCATATGGCTTTAGTGAGGTCTGTTACTGCCTAGGCTTCCCGGCTAGAAGCGGTA 753
```

EXON IV

```
mouse_BDNF      GGGAAGCATATGGCTTTAGTGAGGTCTGTTATTGCCTAGGCTTCCCGGCTAGAAGCAGTA 757
                 * **** * ********* ********* * ***** ******* **** ***   ***

human_BDNF      ACTTGGTGTAACGTTATCTGGGGGCGTTCATCAATAAAAAATGCTGTTATTATCTTGATT 811
rat_BDNF        ACTTGATGTAATGCTATCCGGGGTTATTCATTAATAAAAAATACTGTTATTATCTTGATT 813
mouse_BDNF      ACTTGGTGTAATGCTATCCGGGGTTATTCATTAATAAAAAATACTGTTATTATCTTGATT 817
                ***** ***** * **** ****   ***** ********** *****************

human_BDNF      GAATTCCTATTAGGCAAACTCTAGAGAGGTCAGTGCGCGAACTCTGTTTAAGCCGGCGTG 871
rat_BDNF        GAATTC-TATTAGGCAAACTCCGGAGAGGTCAGGGCGCGAACTCTGGTTAAGCTGATGTG 872
mouse_BDNF      GAATTC-TATTAGGCACACTCCGGAGAGGTCAGGGCGCAAACTCTGGTTAAGCCGATGTG 876
                ****** ******** ****  ********** **** ******* ****** *  ***
                              ▼ ▼▼○
human_BDNF      TTTAAGGCAGCAGAGTAAACCAATAGCCCCCATGCTCTGTGCGATTTCATTGTGTGCTCG 931
rat_BDNF        TTTAAGGTAGCGGAGTAAACCATAACCCCGCACACTCTGTGTAGTTTCATTGTGTGTTCG 932
mouse_BDNF      TTTAAGGTAGCGGAGTAAACCATAACCCCGCACACTCTGTGTAGTTTCATTGTGTGTTCG 936
                ******* *** **********  * *** **  *******   ************ ***
                                                                               EXON V
human_BDNF      CGTTCGCAAGCTCCGTAGTGCAGGAAG]GTGCGGGAAG]GTGTGTCTGTGGCCCGGG----- 986
rat_BDNF        CTTTTTCTAGCTCTGTGGTGCGGGAAGGTGCGGGAAG]GTGTGTCTGGGGCAGGGGC---- 988
mouse_BDNF      CTTTTTCTAGCTTTGTGGTGCGGGAAGGTGCGGGAAG]GTGTGTCTGGGGCCGGGGCCTGG 996
                * **  * ****  ** **** *********************** ***  ***

human_BDNF      ----------AAACGCACGCCCTCTCCCAGAGAACTTGGGTGCTGGGATGGGGAGGAAGG 1036
rat_BDNF        ------GGGGGGATGCACGCCCTCTCCCACAGAACTTGGGTGTTGGGATGGGAAAGATGG 1042
mouse_BDNF      GGGTGGGGGGTGGATGCACGCCCTCTCCCACAGAACTTGGGTGCTGGGATGGAAAAGATGG 1056
                            * ************** *********** ********  * ** **
                  ▼  ▼
human_BDNF      GGAGAGTTGAAAGCTAGGGGAGCGAGACCTCGGGGCGTGCGATTCTCACTCGCTCCCTCC 1096
rat_BDNF        GGAGAGTTGCAAACCAGGGGAGAAAGATTTGGGAGCGTGGTAGTCACACTTGTTCCCTCC 1102
mouse_BDNF      GGAGAGTTGCAAACCAGGGGAGAAAGATTTGAACGTGTGAGACTCACACTCGCTTCCTCC 1116
                ********* ** * *******  ***  *    * ***  * ** **** * * *****

human_BDNF      CGCCCCAGCGCCCACAGCCGGGGTTTCTGCAGAGGGAGAGGGACGCGGGGTTCCCCGGGG 1156
rat_BDNF        TACTCTGGGGCACATTGAGGGGA-----GTAGGGTTCCTAGGAACCGGAATTGGACCACC 1157
mouse_BDNF      TACTCTAGAGCACATTGCGGGGA-----GTAGGGTGCCTAGGAACCGGGATTCGACCACC 1171
                  * *  * ** **  *  ***      * ** *      ***  ***  **   *
                                                                               EXON Vh
human_BDNF      CTGAGGCTGGGGCTGGAACACCCCTCGAAG-CCGCGGGCGTCCTGTCCAAGGCGCCCCAG 1215
rat_BDNF        CCT---CCTAGCCCAGGGCACCCTGCGGAGACAGCAGAGGGCAGGCATAGAGCTTTGGGG 1214
mouse_BDNF      CCT---CCTAGCAAAGGGCACCCTGCGGAGACCGCAGAGGGCAGGCACAGAGCTTTGGGT 1228
                *     *   *    *  *****  ** ** * ** *  *  *   *   *  **

human_BDNF      GAGGGCGCAGGACTCGCAGGGCGATGTCGCGGGGCCCTAGGGGAGGAG]GTGAGGACAGGC 1275
rat_BDNF        TTAAGCAGCTGGTTC-CGGGGAAATGGACAGAAGCCGGTGCAGGGGAATTAGGGATACCC 1273
mouse_BDNF      TTAAGCAGCGAGCTC-CGGGGAAATGGAGAGAAGCCAGTGCAAGGCGATCAGGGATACCC 1287
                    **        ** * *** ***     * ***   *    *       *** *  *

human_BDNF      CCCGGGGGAGCGGGGAGTTCC--GGGCGCCCCTCGGTTTC-CCCGCGCGAGGAAAAGACG 1332
rat_BDNF        CA-------------------AGGGCTTCCGAGGGCTGAATCCCGGCAAGGAAAAGGCG 1313
mouse_BDNF      CGAGGGCTTCCGAGGGCTTCCGAGGGCTTCCGAGGGCTGAGCCCCCGCAAGGAAAAGGCG 1347
                *                      ****  **   ** *    **  ** ******** **

human_BDNF      CGGCGTTCCCTTTAAGCGGCCGCCTCGAACGGGTATCGGTAGCGCGGGCGAGCGGGGAGC 1392
rat_BDNF        CGTCGTCCCCTTTAAGCAGCCGCCCCGAATGGGTATCGGTGGCGCGGCTGAGCAGGGAG- 1372
mouse_BDNF      CGTCGTCCCCTTTAAGCAGCCACCCC-AATGGGTATCGGTGGCGCGGCGGAGCAGGGAG- 1405
                ** *** ********** *** ** * ** ********** ******  **** *****
                                                           ○             ○   ▼▼
human_BDNF      GGGGGGCGGGGGGCGGGGGGGGGGCGGCGCCGTTTGACCAATCGAAGCTCAACCGAAGA 1452
rat_BDNF        ---------------GGGATAGGGGCGGCGCTGTCTGACCAATCGAAGCTCAACCGAAGA 1417
mouse_BDNF      ---------------GGGATAGGGGCGGCGCTGTCTGACCAATCGAAGCTCAACCGAAGA 1450
                               ***   ********** ** ************************
                  ▼▼   ▼○                    ○
human_BDNF      GCTAAATAATGTCTGACCCGGGCGCAAGGCGCAGCCTGGAGCTCCGGGTCCCCGCCGCTG 1512
rat_BDNF        GCTAAATAATGTCTGACCCCAGTGCCTGGCGCTGGCTG-AGCTCTGGGTGCCCGCCGCTG 1476
mouse_BDNF      GCTAAATAATGTCTGACCCCAGTGCCTGGCTCTGGCTG-AGCTCTGGGTGCCCGTCGCTG 1509
                ******************* * **  *** * * *** ***** **** **** *****
```

```
human_BDNF    CCGCCGCCGCGCCCGGGCGCACCCGCCCGCTCGCTGTCCCGCGCACCCCGTAGCGCCTCG 1572
rat_BDNF      CCGCCGC---GCCGGGGCGCACCCGCTGGCTGGCTGTCGCACGGTCCCCATTGCGCCCGG 1533
mouse_BDNF    CTGCCGT---GCCGGGGCGCACCCGCTGGCTGGCTGTCGCACGGTTCCCAGTGCGCCCGG 1566
              * ****    *** ************  *** ****** * **    ***    *****  *

human_BDNF    GGCTCCCGGGCCGGACAGAGGAGCCAGCCCGGTGCGCCCCTCCACCTCCTGCTCGGGGGG 1632
rat_BDNF      GACTCCCCGGCTTGGAGAAGGAAACCGCCTGGGGCGGCGCGCCACCTCC-GCCTGGCAGG 1592   EXON VI
mouse_BDNF    GACTCCCGGGCTTGGAGAAGGAAACCGCCTGGGGCGGCGCGCCACCTCC-GCCTGGGAGG 1625
              * ***** ***  *     ****  * *** ** *** * * ******** **  **  **

human_BDNF    CTTTAATGAGACAC---------------CCACCGCTGCTGTGGGGCCGGCGGGGAGCAG 1677
rat_BDNF      CTTTGATGAGACCGGGTTCCCTCAGCTCGCCACCGCTGCTTTGGGGCAGACGAGAA--AG 1650
mouse_BDNF    CTTTGATGAGACCCGGTTCCTTCAACT-GCCACCACTGCCTTGGGGCAGACGAGAA--AG 1682
              **** *******                ***** ****  ****** * ** * *  **

human_BDNF    CACCGCGACGGGGACCGGGGCTGGGCGCTGGAGCCAGAATCGGAACCACGATGTGACTCC 1737
rat_BDNF      CGC----ACGGGGCCCAGGGCAGGGCGCAGGGACCAGGAGCGTGACAACAATGTGACTCC 1706
mouse_BDNF    CGC----ACGGGGCCCAGGGCAGGGCGCAGGGACCAGAAGCGTGACAACAATGTGACTCC 1738
              * *    ****** ** **** ****** **  **** * **  ** ** **********

human_BDNF    GCCGCCGGGGACCCGTGAG]GTTTGTGTGGACCCCGAG]GTAGGCAAGCGCTGGGAATGGGG 1797
rat_BDNF      ACTGCCGGGGATCCGAGAGCTTTGTGTGGACCCTGAG]GTAGGCGACTGCGGGGCGTGGGG 1766
mouse_BDNF    ACTGCCGGGGATCCGAGAGCTTTGTGTGGACCCTGAG]GTAGGCGACTGCGGGGCGTGGGG 1798
               * ******** *** *** ************ ******** *  ** ***  *****

human_BDNF    CTTGGTGCAGGAGCT----GCCCGTCCGCGGGAGAGAGTTGACTGGGGGATCCCCCACCC 1853
rat_BDNF      CTAAGCGCGAGAGCTGGAGGCGCGTGCGCCCGGGAAAGTTGGCTAGGGGA-------CTG 1819
mouse_BDNF    CTAAGTGCGAGAGCCAGAGGCGCGTGCGCCCGGGAAAGTTGGCTAGGGGA-------CTG 1851
              **  * **  ****      ** *** ***  * ** ***** ** *****        *

human_BDNF    CAAAGTTGTGGGACGAGGCCATGTCTCCTTCTTTCCTCCCCTCCGGTAGAAGGGACGATT 1913
rat_BDNF      AGAAGTTGTGGGACCG---CATAACTTCGGCTTCACCTTCGTCCCGGGACAGCGGAGGG- 1875
mouse_BDNF    AGAAGTTGTGGGACTG---CAGAACTCCGGCTTCACCTTCGTCCCGGGACAGCGGAGGGG 1908
                ************     **    ** *  ***  *   * *** *    ** *  *

human_BDNF    TGGAGTTACTCTTGGGGAGTTTTCTCCCCC-ATCCCACAACCCAGAAGGTCAGCCGGCAC 1972
rat_BDNF      CGGGGTTGCTCCTGAGGAGCGTTCTCCCGGGAGCCCACGACCCCGAGAGACAGC------ 1929
mouse_BDNF    CGGGCTTGCTCCTGGGGAGCGTTCTCCCGGGATCCCACGACTCTGAGGGACAGT------ 1962
               **  ** *** ** ****  *******   * ***** ** * **  * ***

human_BDNF    CACCAGGGAAAAAGGGACCCGGGGAAGTCACGAAGTAGAGGAGGGAAGGCCTGGAGGAGA 2032
rat_BDNF      -------------GCGACCCCGGGAATCCCTTCAGTGCGAGAGAAAAAGCCAAGGGAAGA 1976
mouse_BDNF    -------------GCGACCCCAGGAATCCCTTCAGTGTGAGAGAAAAAGCCAAGGGAAGA 2009
                           * *****  ****  *    ***    ***  ** ***  * * ***

human_BDNF    CCCAGAGCTGCGTGATGGGAGCAAAGACGGCGACCCGGGGATCCCTCGCAGCCCTCCCCC 2092
rat_BDNF      GGACGACTTGCGCCATGGGAGCAAAGCCTGCGTCCCGGGGATCCC-CGAAGTCCTCCCGC 2035
mouse_BDNF    GGACGACTTGCGCCATGGGAGCTAAGCCTGCGTCCCGGGGATCCC-CGAAGTCCTCCCGC 2068
                 **  ****  ******** *** * *** ************ ** ** ****** *

human_BDNF    AGCCCAGGAGTAGTCGAGAGAGACTTAGGGGGCCAGAGCTGTCGAGGGTCCTGACTGAGG 2152
rat_BDNF      AGACGAGGAAGGGTGGTGAGAGAGATGTAGGGCAACAGCTGTCGGGGGGCTCAGACGGAAG 2095
mouse_BDNF    AGACGAGGAAGGGTGGTGAGAGAGATATAGAGCGACAGCTGTTGGGGGGCTCAGACGGATG 2128
              ** * ****    ** * ******  *    * ** * ****** * ***  * *** ** *

human_BDNF    GGAGGGTGCTGGGGCTAGGCTAGGAATCCTT-CCAGGGGGT-GGGTGGTCCCCGCGCCGA 2210
rat_BDNF      GCACCGAGTCTGGGCTGGGATGAGATCCCTTACCACAGGCT-AGGCACTCTCCACGCAGA 2154
mouse_BDNF    GCACCGGGACAGGGCTGGGATGAGAGCCCTTACCACCGGCTTAGGCACTCTCCACGCAAA 2188
              * *  * *   ***** ** *  **  **** *** ***  ** *  **    ** ** ***  *

human_BDNF    CTTG---CGGGGGG------AGT-------GGGAGGGAAGCTTG-CGCCTTCAGCCCGCA 2253
rat_BDNF      TTTGGACCGGGGTGGTGTGTAGTGTAGTCCGAGAATGGGTCTTGGCGCCCTCTGCCCTCA 2214
mouse_BDNF    TTTGGACCGGGGTGGTGTGTAGT-----CCGAGAATGGGTCTTGGCGCCTTCTGTCCTCA 2243
               ***    ***** *       ***        * **  *    **** **** ** * ** **

                                                   ▼○
human_BDNF    TCCCTTCCCCGGA------------GCTGCACACGGCTACCTGCTCCC-AGGAATTGAGA 2300
rat_BDNF      TCTCTTTTCCTGGGTATCACAACAGGCTGTTCACTGTCACCTGCTTTCTAGGGAGTATTA 2274
mouse_BDNF    TCTCTTTTCCTGGGTATCACAACAGGCTGTTCACTGTCACCTGCTCTCTAGGGAGTACTA 2303
```

```
                 ** ***   ** *              ****   *** *  *******  * *** * *    *

human_BDNF      CTGAAGTGGACTTACAAGTCCGAAGCCAATGT--AGCTTGGAAAACTTGGGAGGCGGAAT 2358
rat_BDNF        CCAAAGCTT-CTTACAAGTCCAAGGTCAACAT--AACTAGGAA--CTTGGGATCA----T 2325
mouse_BDNF      CCAAAGCTTACTTACAGGTCCAAGGTCAACGTTTAACTAGGAAAACTTGGGACCC----T 2359
                *  ***     ****** **** * * ***   *   * ** ****   *******      *

human_BDNF      TCCTACCGCTGGGAACTGAAAGGGTCTGCGACACTCTCGG----GCAGGCCGAACCCACA 2414
rat_BDNF        TCTTGTCACTGGGACCTGAAAGGGTCTGCGGAACTCCAGGTCCCACAGCTTGTATCCGAC 2385
mouse_BDNF      TCTTATCGCTGGGGCCTGAAAGGGTCTGCGGAACTCCAGG----ACAGCCTGTATCCGAC 2415
                ** *  * *****  **************  ****  **      ***    * * **

human_BDNF      TCTCT--ACCCATCCTGCGCCCCTCTTCTGAAGCGCCCTCCAGGGAAGTTAAGAGTTTTG 2472
rat_BDNF        CCTCTCTGTCCATCCAGCGCACCTC--TTTAGGCATCCTCCAGAGGAAGTGAAAGTTTTG 2443
mouse_BDNF      CCTCT--GTCCATCCTGCGCACCTCCCTTTGGGCATCCTCCTGAGGAAGTGAAAGTTTTG 2473
                 ****     ****** **** ****    *    **   ***** * * *   * * *******

human_BDNF      ACTTTCG---GGGAGTGGTTGGGATGTACGTGGGGGATTCTT-GACTCGGGTTAGTCTCT 2528
rat_BDNF        ACTTTCATCCGGGAGTAGATTGGGTGTCTGTGGAGGATGAAGAGTCTGGTTTTGGTTTCT 2503
mouse_BDNF      ACTTTCATCCGGGAGTAGGTTGGGTGTTTATGGAGGATGAAGAGGCTAGTTTTGATTTCC 2533
                ******     ****** * * ** ***    *** ****      * ** *   **   * **

human_BDNF      GGGGATGCAGAGCCGGGAAGAGGAATGGGTGAGTGAGTTACTCCTGGAAAGAAATAGCTG 2588
rat_BDNF        GGAGATGCTGGAACAGGATGGGGAATGGATGAGCGACCTAAGCGTAAAAAGAAATAGCTG 2563
mouse_BDNF      AGGGATGCTGAAACAGGAAGAGCAATGGATAAGCGACCTAAGCGTAGAAAGAAATAGCTG 2593
                 * ***** *    * ***  * * ***** * ** **   **   * *   ************

human_BDNF      AGGATTGGGGGCTCTGTGCCTGACGGGCAAGAAGAAGGGGAGATTACAGACTAGGGGCAT 2648
rat_BDNF        AGGAT-GCCAGCTTTGTGCTTGATGACAAGGAAGAAGGGGAAAATGCTGCCCGGGGGGTT 2622
mouse_BDNF      AAGAT-GGGGGCTTTGTGCTTGATGACAAGGAAGAAGGGGAAATTGCTGCCGGGGGTGGG 2652
                * *** *    *** ***** *** *    * *********** * * * * *   ***

human_BDNF      CCCTAAGGAAGA 2660
rat_BDNF        CCCCCGG----- 2629
mouse_BDNF      GGGGGGGTG--- 2661
                      *
```

< ~ 25 kb >

```
human_BDNF      GCAGTGGCTAGGAGAACATATTCAATTATATTTCCTCCTGCATTAAGCTACAAGTAATGA 60
rat_BDNF        GCAGTGGGCAGGAAGACATATT---TTATTTTTCCTCCCACATTAAGCTGCAAATAAGGA 57
mouse_BDNF      GCAGTGGGCAGGGAGACATATT---TTATTTTTCCTCCCACATTAAGCTGCAAATAATGA 57
                *******  ***    *******    **** ********  ********* *** *** **
                                                 ○        ○
human_BDNF      GCACTTTCCTGTGCTTTACAGTTAAGTAATTAAAAGAAATTATAGAGTGGGATGCAAAAA 120
rat_BDNF        GCGCTTTCCTGTACATTGCAGT-AAGCAATTAAAAGAAGTTATAGAGTTGGATGCAAGCG 116
mouse_BDNF      GCGCTTTCCTGTGCATCACAGT-AAGCAATTAAAAGAAATTATAGAGTTGGATGCAAGCA 116
                ** ********* * *  **** *** *********** ********* ********

human_BDNF      TAACCCGAAGGACAACTGGATGTGTGGAGCCACCAGTTTTCTCCATGAGTGCACAAGGTT 180
rat_BDNF        TAACCCGAAGGACAACTGGATGTGTGGAAGCACCAGTTTTCTCCATGTGCTCA---GGCT 173
mouse_BDNF      TAACCCGAAGGACAACTGGATGTGTGGAACCACCAGTTTTCTCCATGTGCTCA---GGCT 173
                ****************************  ***************** *  **    ** *

human_BDNF      AATCCTTGTTACTA-CTCAGAATGCTGAGTTTCTACAGAAAGGGTTGCAGGTCCACACAT 239
rat_BDNF        AATCCTCGTTAATA-CTCAGAACGTTGAGTTTCTACGGAATGGGTTGCATGTCCATACAT 232
mouse_BDNF      AATCCTCATTAATAACTCAGAAAGTTGAGTTTCTACAGAATGGGTTGCATGTCCATACAT 233
                ******   *** ** ******* * *********** *** ******** ***** ****

human_BDNF      GTTTTGGCGTCTACCCACACGCTTCTGTATGGCATGACTGTGCATCCCAGAAGAAGGGCT 299
rat_BDNF        GTTTTGGTATCTACCCACACACTTTCATGTGGTATGACTGTGCATCCCAGGAGAAAGGCT 292
mouse_BDNF      GTTTTGGCATCTACCCACACACTTTTATGTGGTATGACTGTGCATCCCAGGAGAAAGGCT 293
                *******  *********** ***    * *** **************** **** ****

human_BDNF      GTGCTGTGTACCTCCACGTTTCAGTGGAATTTAACAAACTGATCCCTGAAAATGGTTTCA 359
rat_BDNF        GTGGTGTGTGTCTCTGCGCCTCAGTGGAGCTGAACAAAC-GATTGCTGAAAATGGTGTCA 351
```

EXON VII

EXON VIII

```
mouse_BDNF      GTGGTGTGTGTCGCTGCGCCTCAGTGGAGCCGAACAAACTGATTGCTGAAAATGGTGTCG 353
                *** *****  * *  **  ********    ******* ***  *********** **

human_BDNF      TAAAGGTGAGTAACAGAGAGCTAATAGCCTTCTCTTGCTAATTTTATCTTT-----CCCC 414
rat_BDNF        TAAAGGTGAGCAACAAAGAACTAGTAACCTTCTCTTGCTAATTTTATCCCCTCCACCCT 411
mouse_BDNF      TAAAGGTGAGCAACAAAGAACTAATAACCTTCTCTTGCTAATTTTATCTCCTCCAACCCT 413
                ********** **** *** *** ** ********************         ***

human_BDNF      CAAGATTTCTTGATAATAGTTTGAAAAGGAGTGTTATTCTTTGGTCTCTAGAGGCAACTT 474
rat_BDNF        CAGGACATCGTGAGAGTAATTTGAAAAGGATTGTTGTTCTTCTGTTGCCAGGGGCAACTC 471
mouse_BDNF      -GGGACATCGTGATAGTAATTTGAAAAGGAATGTTGTTCTTCTGTTGCTAGGGACAACTT 472
                   **  ** *** * ** *********** **** *****  **   * ** * *****

human_BDNF      ACCTT 479
rat_BDNF        ATCTT 476
mouse_BDNF      ATCTT 477
                * ***

< ~ 13,5 kb >

human_BDNF      GTGCAATCTAAGCAGTTCTAAGCAT--GTTTGCTATTCGTGCAAAG-TGAGAGTAAATCT 57
rat_BDNF        GGATCATTGCTGTCTTCTCGTTGATTGGCTACCCCTACCGTCCAAG-CAGTTCTGAGTAT 59
mouse_BDNF      CTCTTACTGTGGCTGATGTGTGGA--GGCTGTGCACATATACGAAGTCGGAAACTGTTGT 58
                     *     *           *   * *           * ***           * *

human_BDNF      AAAAGAAATTTTTTTGTGT------GTTTAGGGATGGTAATAAAGTCTCTTAGTGGTTGA 111
rat_BDNF        GTTTACCATTTATGCAAACTGAAG-GTGTATACATGAGGAAATTTCCATTG-GTGGTAGA 117
mouse_BDNF      GTGCATTGCTCTCCTGAGGGGAAGAGTCTCAAGATAATGGTGATGCCACTGTAGAATTGG 118
                         *               ** *    **            *  *      * *

human_BDNF      AAATGTTATTTCTTACAAAAGTGGAGAACATTTGCTTTTCAATACCAGAGTTTTCAGCCA 171
rat_BDNF        CTACCTTGTCTCTTTCAAAAGTGGATGATAGAAACTTTGATTTTTCAATACTAA-AGCTT 176
mouse_BDNF      AGAGAGCTTTAATGTCCCACTTTGGCAAGAATTCCCATAGCTTGT-GTTGCGTA-AGCTG 176
                  *     *   *  *  *  * *   * *      *  *    *          ***

human_BDNF      TTTCTGCATTCTGACCTATTG-ACTGGAGGTAGGTTGCCTTTGAATTCAGTAAAACTTCA 230
rat_BDNF        TCGGCCATTTCTGCACTATTG-ACTGGAGGTAGGTTATTTTTGTGTTCAGCAACTCATCG 235
mouse_BDNF      TCTGTGCACTGTGCCCTATTTCACTGGAGGTAGGTTGTTCTTGTGTTCAGCATCTCATTG 236
                *        * **  *****  *************     ***  ***** *   * *

human_BDNF      TGGGCAGAAACACAGTTCCTTTTCCTACTTATTTGGATATCATGATGGCCATTGCATGTA 290
rat_BDNF        TGGACAGAAACATAATTCTTTT-CTGGTGGACTTACCCTGCATAATGAGTGCTGTGCGTG 294
mouse_BDNF      TGAGCAGAAACATGCTTCCTT--CTGGTGGACTTACCCTGCATAATGAATGCTGAGTGTG 294
                **  ********   *** **   *      * **       *** ***       **   **

human_BDNF      TGTGTCTTTTTGTAAGTCCATGCCTCAGAACTGAGAAGTAGGAATAAAATTAGG--GTCA 348
rat_BDNF        TATGTCTTT--ACGAGTCTATGTTCCACAGCTGATAAGTAGAAAGAAAGTTAGCACGGTA 352
mouse_BDNF      TATGTCTTT--ACAAGTCTATGTTCCACAGCCAATAAGTAGAAAGAAAGTTAGCCTATTA 352
                * *******     **** ***   ** *  *  ****** ** *** ****      *

human_BDNF      GGGCT--GGGGATGCTACTCTTTGCTGCTGAGAAACACAATGCTTCAGGTAAGTGATTCT 406
rat_BDNF        GAGTTAAAGGAATTCTACTTGTTACTACTAAGAGGCACAATGCTTCCGGTAAGTAACTCA 412
mouse_BDNF      GAGTTAAAGAGATGCTACTCGTTACTACTAAGAGGCAGAATGCTTCCGGTAAGTAATTCA 412
                * * *   *  ** *****  ** ** ** ***   ** ******** ******* * **

human_BDNF      GAAGTCCTTCACCACCTGACGGTAACCTTGGGTTGGTCCATAGGTATGTTTTCATTTTGC 466
rat_BDNF        GAAGTCTTCCACCACCTGACG-TAACCTTGGTTTGGTCCATAGGTATGTCTTAATTTTGC 471
mouse_BDNF      GAAGTCTTCCACCACCTGATG-TAAGCTTGGTTTGGTCCATAGGTATGTGTCAATTTTGC 471
                ****** * ********** * *** ***** **************** *  *******

human_BDNF      TTGTTCATCCATTTTAATTGGCTTCCTAGAGCATGCTTGTAGATGTAGAGCCAAATTTAG 526
rat_BDNF        TTCTTATTCAATTTTAGTTGGGTTTGCAGAGCGTGCTTAAAGATGCA-AGCCAAATTC-- 528
mouse_BDNF      TTGTTATTCAATTTTAGTTGGGTTTGCGGAGTATGCTTATAGATGCA-AGCCAAATT--- 527
                ** **  ** ****** **** **     ***  *****  ***** * *********
```

EXON VIIIh

```
human_BDNF     AGTAGAGCAACCCTCTGGCAAACAGGAAGAGATTAATTTTGTGGTATGCTTTTAAGGGAC 586
rat_BDNF       ----------------------TAAGAGTGATTTAACTTTGTGGTATGTTGTTAAGAGGC 566
mouse_BDNF     ---------------------------------TAACTTTGAGGTATGTTATTAAGTGAC 554
                                                *** **** ****** * ***** * *

human_BDNF     TTCCCAGGAAACTTCAAAAGCAGAAAAAGAAGCACTAGCTGCCTATTCCAAAATGTGTAA 646
rat_BDNF       ATCATAGGGAACTTCAAATGCACAGAAAGAAACCCTAGCTGCCAGTTCCAAAGTGTGTAA 626
mouse_BDNF     TCTGTAGGAAAGTTCAAATGCACAGAAAGAAGTACTAGATGCCAATTCAAAAGCGTGTAA 614
                    *** ** ****** *** * ******   **** ****  *** ***  ******
                                              ▼
human_BDNF     AACACCACTCAGCTTTTTAAAAGTAGGATAAACTCAGAG---CGGACACACACACACACA 703
rat_BDNF       AATACTACTCAGCTTTGTAAAGGTATGACAA-CTCAGTGTTACACACACGCGCGCGTGCA 685
mouse_BDNF     AGTACTACTCAGCTTTGTAAAGGTATGACAA-CTCAGTGTTACACACACACACACACACA 673
               *  ** ********** **** *** ** ** ***** *   *  **** * * *   **

human_BDNF     CACACACAGAGAGA-ACATCTCTAGTAAAAAGAAAAGTTGAGCTTTCTTAGCTAGATGTG 762
rat_BDNF       CACACACACACACACACA--------------------GGAGTTCTGCTAATGAGAAGT- 724
mouse_BDNF     CACACACACACACACACACACACACTTGAGAAGGGGGTGGAGTTCTGCTAATGAGAAGC- 732
               ******** * * * ***                     *** * *  **   *** *

human_BDNF     TGTATTAGCCAGAAAAAGCCAAGGAGTGAAGGGTTTTAGAGAACTGGAGGAGA-TAAAGT 821
rat_BDNF       TGCGCTCTCTTTGGTTTGCCAAAAC----AAGCCATCCAAGTACCATGGGAGTCTGGAGA 780
mouse_BDNF     TGAGCTCTCTGTGGTTTGCCAAAAC----AAGCCATCAAAGTGCCCTCTGAGTCTGGAGA 788
               **   *  *        *****       * *   *   **  *     ***  *  **

human_BDNF     GGAGTCTGCATATGGGAGGCATTTGAA---ATGGACTTAAATGTCTTTTTAATGCTGACT 878
rat_BDNF       GATGTCCGCACGTGAAAGGCATTGATTGATCCGGACTTGAATGCCTTTCCT-TGAGGACT 839
mouse_BDNF     GATGTCTGCTCGTGAAAGGCATTGAT----CCTGACTTGAATGCCTTTCCT-TGAGGACT 843
               *  *** **   **  *******          ***** **** ****    **  ****

human_BDNF     TTTTCAGTTTTCTCCTTACCAGACACATTGTTTTCATGACATTAGCCCCAGGCATAGACA 938
rat_BDNF       AGTCCAGCTTTCCC-CTTTCT--CTT------------GTTTTCATACCTGCCTCAGGCA 884
mouse_BDNF     GTTCCAGCTTTCCAGCTTTCT--CTTAAAAGATACATTGTTTTCATCCCTGCCTCAGGCA 901
                 * *** ****    *  *   *                 **    ** * *  ** **

human_BDNF     CATCATTAAAATGAACATGTCAAAAAATGATTTCTGTTTAGAAATAAGCAAAACATTTTC 998
rat_BDNF       CATCATTAGAACAAACCTACTGAAAACGAATT---GTTTGGAAATAAGCAAAACCTTCTC 941
mouse_BDNF     TGTCATTAGAACAAACCTATTGAAAATGAATT---GTTTGGAAATAAGCAAAACCTTCAC 958
                 ****** **  *** *    ****   ***   **** ************** **  *

human_BDNF     AGTTGTGACCACCCAGGTGTAGAATAAAGAACAGTGGAATTGGGAGCCCTGAGTTCTAAC 1058
rat_BDNF       AGATGCAGT------------------AAAAC-CTAGAGATGTGCTCTCT-AGTTCC--- 978
mouse_BDNF     AGATGTAGT------------------AAAACGTTGGAGATGGGATCTCT-AGTTCC--- 996
               ** **                      * ***  * **  ** *  * ** *****

human_BDNF     ATAAACTTTCTTCATGACATAAGGCAAGTCTTCTATGGCCTTTGGTTTCCTTACCTGTAA 1118
rat_BDNF       -TAAAGTTTCTTCATGACACCAGACAAATCCTGTTTGGCCTTTAGCTTCCCTATCTGAAA 1037
mouse_BDNF     -TTAAGTTTCTTCATGAGACCGGGCAAGTCCTGTGTGGCCTTTTGGTTCCTTATCCGCAA 1055
                * ** *********** *   * *** ** * * ******** * **** ** * * **

human_BDNF     AACAGGATGGCTCAATGAAATTATCTTTCTTCTTTGCTATAATAGAGTATCTCTGTGGGA 1178
rat_BDNF       AACACCGTGGTCCGAA-----------GTGCTGTCACACTG--------CCTCTAGGGAG 1078
mouse_BDNF     AACATGGTGGTCTACA-----------TCGCCTCTAGGAGGAAAAGGCCCCTCCCAGCAT 1104
               ****   ***                                        ***   *

human_BDNF     AGAGGAAAAAAAAAGTCAATTTAAAGGCTCCTTATAGTTCCCCAACTGCTGTTTTATTGT 1238
rat_BDNF       AAAGG------------------------------------CCCGTCCCAGCT------- 1095
mouse_BDNF     TCCGG------------------------------------CCGACCGCTGTTTTATCAT 1128
                  **                                    **     * * *
                                                                      ○
human_BDNF     GCTATTCATGCCTAGACATCACATAGCTAGAAAGGCCCATCAGACCCCTCAGGCCACTGC 1298
rat_BDNF       ----CTCCTGCTCAGA---------GCTGCAGCCAGCCGGC-----CCTCTAGCCACTAC 1137
mouse_BDNF     ACTGCTCCTGCTCAGACTGCTTAGAGCACCAGACACCCAGC-----CCTCTAGCCACTAC 1183
                    ** ***  ***         **   *     **  *     ****  ****** *

human_BDNF     TGTTCCTGTCACACATTCCTGCAAAGGACCATGTTGCTAACTTGAAAAAAATTACTATTA 1358
rat_BDNF       TGTTTCCACCACACATTCCTACCAAGGACTGTGCTGCTGACTTGAAAGGAAG-ATTACTA 1196
mouse_BDNF     CGTTTCCATCACACATTCCTCCCAAGGACTATGCTGCTGACTTGAAAGGAAG-ATTACTA 1242
```

```
                       *** *   *********** * ******  ** **** ********  **  * ** **
                                                                          ○
human_BDNF           ATTACACTTGCAGTTGTTGCTTAGTAACATTTATGATTTTGTGTTTCTCGTGACAG[CATG 1418
rat_BDNF             -TTCCACTTGCAGTTGTTGCTTAGTAACATGTGTGATT--GTGTTTCTGGTGACGGCGTG 1253
mouse_BDNF           -TTCCACTTGCAGTTGTTGCTTAGTAACATTTGTGATT--GTGTTTCTGGTGACAGCGTG 1299
                      ** ************************** * *****  ******** ***** ** **

human_BDNF           AGCAGAGATCATTAAAAATTAAACTTACAAAGCTGCTAAAGTGGGAAGAAGGAGAACTT- 1477
rat_BDNF             AACAGAGATCATTAAAAATTAAACCCCCAGAGCTGCTAAAGTGGGAGGAAGTGGGAGCGG 1313
mouse_BDNF           TGCAGAGATCATTAAAAATTAAACCCCCAAAGCTGCTAAAGCGGGAGGAAGTGGGAGCAG 1359
                       *********************    **  *********** **** ****  * *

human_BDNF           -GAAGCCACAATTTTTGCACTTGCTTAGAAGCCATCTAATCTCAG]-TTATATGCTAGAT 1535
rat_BDNF             GGAAGCCACAGTGCCGTCACTTGCTTAGAAACCGTTTACTCTCAGGCTTACATG-TAAAT 1372
mouse_BDNF           GGAAGCCACAGTGTGTTCACTTGCTTAGAAACCGTTTACTCTCAGGCTTACATG-TAAAT 1418
                      ********* *      ************* ** * ** ******* *** *** ** **

human_BDNF           CTTGGGGGCAAACACTGCATGTCTCTGGTTTATATTAAACCACATACAGCACACTA-CTG 1594
rat_BDNF             CTTGAGGA-AAACACTGCATGTCTCTGGTTTATATTAAGCTACATACAGCACGGTAACTG 1431
mouse_BDNF           CTTGGGGA-AAACATTGCATGTCTCTGGTTTATATTAAGCCACATACAGCACAGTAACTG 1477
                     **** **  ***** ********************** * **********   ** ***

human_BDNF           ACACTGATTTGTGTCTGGTGCAGCTGGAGTTTATCACCAAGACAT------------AAA 1642
rat_BDNF             ACATTGATTTGTGTCCCCTGCAGCTGGAGTGGATCAGTAAGACATTAAAAAAAAAAGAAA 1491
mouse_BDNF           ACATTGATTTGTGTCCCCTGCAGCTGGAGTGGATCAGTAAGACA------------AAA 1524
                     *** ***********   ***********  ****  ******             ***

human_BDNF           AAAACCTTGACCCTGCAGAATGGCCTGGAATTACAATCAGATGGGCCACATGGCATCCCG 1702
rat_BDNF             AAGAAAAAAAGAAAGAAAAAAGGTCTGAAATTACAAGCAGATGGGCCACATGGTGTCCCC 1551
mouse_BDNF           AAAAAAAAAAAAAAAAAAAAAGGTCTGAAATTACAAGCAGATGGGCCACATGCTGTCCCC 1584
                     ** *      *      * ** ** *** ******** ***************   ****

human_BDNF           GTGAAAGAAAGCCCTAACCAGTTTTCTGTCTTGTTTCTGCTTTCTCCCTACAG[TTCCACC 1762
rat_BDNF             AAGAAAGTAAGGTCTAACCTGTTCTGTGTCT-GTCTCTGCTTCCTTCCCACAG[TTCCACC 1610
mouse_BDNF           GAGAAAGAAAGTTCTAACCTGTTCTGTGTCT-GTCTCTGCTTCCTTCCCACAG[TTCCACC 1643
                       ***** ***  ****** *** * ***** ** ******* ** ** ***********
                                 →
human_BDNF           AGGTGAGAAGAGTGATGACCATCCTTTTCCTTACTATGGTTATTTCATACTTTGGTTGCA 1822
rat_BDNF             AGGTGAGAAGAGTGATGACCATCCTTTTCCTTACTATGGTTATTTCATACTTCGGTTGCA 1670
mouse_BDNF           AGGTGAGAAGAGTGATGACCATCCTTTTCCTTACTATGGTTATTTCATACTTCGGTTGCA 1703
                     **************************************************** *******

human_BDNF           TGAAGGCTGCCCCCATGAAAGAAGCAAACATCCGAGGACAAGGTGGCTTGGCCTACCCAG 1882
rat_BDNF             TGAAGGCTGCGCCCATGAAAGAAGCAAACGTCCACGGACAAGGCAACTTGGCCTACCCAG 1730
mouse_BDNF           TGAAGGCGGCGCCCATGAAAGAAGTAAACGTCCACGGACAAGGCAACATGGCCTACCCAG 1763
                     ******* ** ************* **** *** ** ********   * ************

human_BDNF           GTGTGCGGACCCATGGGACTCTGGAGAGCGTGAATGGGCCCAAGGCAGGTTCAAGAGGCT 1942
rat_BDNF             CTGTGCGGACCCATGGGACTCTGGAGAGCGTGAATGGGCCCAGGGCAGGTTCGAGAGGTC 1790
mouse_BDNF           GTGTGCGGACCCATGGGACTCTGGAGAGCGTGAATGGGCCCAGGGCAGGTTCGAGAGGTC 1823
                      ***************************************** ********* *****

human_BDNF           TGAC------ATCATTGGCTGACACTTTCGAACACGTGATAGAAGAGCTGTTGGATGAGG 1996
rat_BDNF             TGACGACGACGTCCCTGGCTGACACTTTTGAGCACGTGATCGAAGAGCTGCTGGATGAGG 1850
mouse_BDNF           TGACGACGACATCACTGGCTGACACTTTTGAGCACGTCATCGAAGAGCTGCTGGATGAGG 1883
                     ****       **  ************* ** ***** ** ********* *********

human_BDNF           ACCAGAAAGTTCGGCCCAATGAAGAAAACAATAAGGACGCAGACTTGTACACGTCCAGGG 2056
rat_BDNF             ACCAGAAGGTTCGGCCCAACGAAGAAAACCATAAGGACGCGGACTTGTACACTTCCCGGG 1910
mouse_BDNF           ACCAGAAGGTTCGGCCCAACGAAGAAAACCATAAGGACGCGGACTTGTACACTTCCCGGG 1943
                     ******* *********** ********* ********** *********** *** ***

human_BDNF           TGATGCTCAGTAGTCAAGTGCCTTTGGAGCCTCCTCTTCTCTTTCTGCTGGAGGAATACA 2116
rat_BDNF             TGATGCTCAGCAGTCAAGTGCCTTTGGAGCCTCCTCTGCTCTTTCTGCTGGAGGAATACA 1970
mouse_BDNF           TGATGCTCAGCAGTCAAGTGCCTTTGGAGCCTCCTCTACTCTTTCTGCTGGAGGAATACA 2003
                     ********** ************************** **********************

human_BDNF           AAAATTACCTAGATGCTGCAAACATGTCCATGAGGGTCCGGCGCCACTCTGACCCTGCCC 2176
```

EXON IX

```
rat_BDNF         AAAATTACCTGGATGCCGCAAACATGTCTATGAGGGTTCGGCGCCACTCCGACCCCGCCC 2030
mouse_BDNF       AAAATTACCTGGATGCCGCAAACATGTCTATGAGGGTTCGGCGCCACTCCGACCCTGCCC 2063
                 ********** ***** *********** ******** *********** ***** ****

human_BDNF       GCCGAGGGGAGCTGAGCGTGTGTGACAGTATTAGTGAGTGGGTAACGGCGGCAGACAAAA 2236
rat_BDNF         GCCGTGGGGAGCTGAGCGTGTGTGACAGTATTAGCGAGTGGGTCACAGCGGCAGATAAAA 2090
mouse_BDNF       GCCGTGGGGAGCTGAGCGTGTGTGACAGTATTAGCGAGTGGGTCACAGCGGCAGATAAAA 2123
                 **** ***************************** ******** ** ******** ****

human_BDNF       AGACTGCAGTGGACATGTCGGGCGGGACGGTCACAGTCCTTGAAAAGGTCCCTGTATCAA 2296
rat_BDNF         AGACTGCAGTGGACATGTCCGGTGGGACGGTCACAGTCCTGGAGAAAGTCCCGGTATCAA 2150
mouse_BDNF       AGACTGCAGTGGACATGTCTGGCGGGACGGTCACAGTCCTAGAGAAAGTCCCGGTATCCA 2183
                 ******************* ** ***************** ** ** ***** ***** *

human_BDNF       AAGGCCAACTGAAGCAATACTTCTACGAGACCAAGTGCAATCCCATGGGTTACACAAAAG 2356
rat_BDNF         AAGGCCAACTGAAGCAATATTTCTACGAGACCAAGTGTAATCCCATGGGTTACACGAAGG 2210
mouse_BDNF       AAGGCCAACTGAAGCAGTATTTCTACGAGACCAAGTGTAATCCCATGGGTTACACCAAGG 2243
                 **************** ** ***************** ***************** ** *

human_BDNF       AAGGCTGCAGGGGCATAGACAAAAGGCATTGGAACTCCCAGTGCCGAACTACCCAGTCGT 2416
rat_BDNF         AAGGCTGCAGGGGCATAGACAAAAGGCACTGGAACTCGCAATGCCGAACTACCCAATCGT 2270
mouse_BDNF       AAGGCTGCAGGGGCATAGACAAAAGGCACTGGAACTCGCAATGCCGAACTACCCAATCGT 2303
                 **************************** ******** ** ************** ****

human_BDNF       ACGTGCGGGCCCTTACCATGGATAGCAAAAAGAGAATTGGCTGGCGATTCATAAGGATAG 2476
rat_BDNF         ATGTTCGGGCCCTTACTATGGATAGCAAAAAGAGAATTGGCTGGCGGTTCATAAGGATAG 2330
mouse_BDNF       ATGTTCGGGCCCTTACTATGGATAGCAAAAAGAGAATTGGCTGGCGATTCATAAGGATAG 2363
                 * ** *********** ***************************** *************

human_BDNF       ACACTTCTTGTGTATGTACATTGACCATTAAAAGGGGAAGATAGTGGATTTATGTTGTAT 2536
rat_BDNF         ACACTTCCTGTGTATGTACACTGACCATTAAAAGGGGAAGATAGTGGATTTATGTTGTAT 2390
mouse_BDNF       ACACTTCCTGTGTATGTACACTGACCATTAAAAGGGGAAGATAGTGGATTTATGTTGTAT 2423
                 ******* ************ ***************************************

human_BDNF       AGATTAGATTATATTGAGACAAAAATTATCTATTTGTATATATACATAACAGGGTAAATT 2596
rat_BDNF         AGATTA-----TATTGAGACAAAAATTATCTATTTGTATATATACATAACAGGGTAAATT 2445
mouse_BDNF       AGATTA-----TATTGAGACAAAA-TTATCTATTTGTATATATACATAACAGGGTAAATT 2477
                 ******     ************* ***********************************

human_BDNF       ATTCAGTTAAGAAAAAAATAATTTTATGAACTGCATGTATAAATGAAGTTTATACAGTAC 2656
rat_BDNF         ATTCAGTTAAGAAAAAGATAATTTTATGAACTGCATGTATAAATGAAGTTTATACAGTAC 2505
mouse_BDNF       ATTCAGTTAAGAAAAA-ATAATTTTATGAACTGCATGTATAAATGAAGTTTATACAGTAC 2536
                 **************** *******************************************

human_BDNF       AGTGGTTCTACAATCTATTTATTGGACATGTCCATGACCAGAAGGGAAACAGTCATTTGC 2716
rat_BDNF         AGTGGTTCTACAATCTATTTATTGGACATATCCATGACCAGAAAG-AAACAGTCATTTGC 2564
mouse_BDNF       AGTGGTTCTACAATCTATTTATTGGACATATCCATGACCTGAAAGGAAACAGTCATTTGC 2596
                 ***************************** ********* *** * **************

human_BDNF       GCACAACTTAAAAAGTCTGCATTACATTCCTTGATAATGTTGTGGTTTGTTGCCGTTGCC 2776
rat_BDNF         GCACAACTTTAAAAGTCTGCATTACATTCCTCGATAATGTTGTGGTTTGTTGCCGTTGCC 2624
mouse_BDNF       GCACAACTTTAAAAGTCTGCATTACATTCCTCGATAATGTTGTGGTTTGTTGCCGTTGCC 2656
                 ********* ********************* ****************************

human_BDNF       AAGAACTGAAAACATAAAAAGTTAAAAAAAA----TAATAAATTGCATGCTGCTTTAATT 2832
rat_BDNF         AAGAATTGAAAACA--AAAAGTTAAAAAAAA----TAATAAATTGCATGCTGCTTTAATT 2678
mouse_BDNF       AAGAATTGAAAACA--AAAAGTTTAAAAAAAATAATAATAAATTGCATGCTGCTTTAATT 2714
                 ***** ********  ******* *******    *************************

human_BDNF       GTGAATTGATAATAAACTGTCC-TCTTTCAGAAAACAGA---AAAAAACACACA-CACAC 2887
rat_BDNF         GTGAATTGATAATAAACTGTCCCTCTTTCAGAAAACAGAC-AAAAAAACAAAAAACAAAA 2737
mouse_BDNF       GTGAATTGATAATAAACTGTCCCTCTTTCAGAAAACAGATTAAAAAAACAAAAAACAAAA 2774
                 ********************** ****************   ******** * * ** *

human_BDNF       A----------------CAACAAAAATTTGAACCAAAACATTCCGTTTACATTTTAGACA 2931
rat_BDNF         A---------AAAGCAAAAACAAAAATTTGAACCAAAACATTCCGTTTACATTTTAGACA 2788
mouse_BDNF       AAAAAAACAAAAAACAAAAACAAAAATTGGAACCAAAACATTCCGTTTACATTTTAGACA 2834
                 *                 ********** *******************************
```

```
human_BDNF      GTAAGTATCTTCGTTCTTGTTAGTACTATATCTGTTTTACTGCTTTTAACTTCTGATAGC 2991
rat_BDNF        CTAAGTATCTTCGTTCTTGTTAGTACT----CTGTTCTACTGCTTTCAACTTCTCATAGC 2844
mouse_BDNF      CTAAGTATCTTCGTTCTTGTTAGTACT----CTGTTTTACTGCTTTCGACTTCTGATAGC 2890
                 **************************    ***** *********  ****** *****

human_BDNF      GTTGGAATTAAAACAATGTCAAGGTGCTGTTGTCATTGCTTTACTGGCTTAGGGGATGGG 3051
rat_BDNF        GTTGGAATTAAAACTATGTCAAGGTGCTGTTGTCATTGCTTTACTGGCTTAGGGGATGGG 2904
mouse_BDNF      GTTGGAATTAAAACAATGTCAAGGTGCTGTTGTCATTGCTTTACTGGCGTAAGGGACGGG 2950
                ************** ********************************* ** **** ***

human_BDNF      GGATGGG--GGGTATATTTTTGTTTGTTTTGTGTTTTTTTT---TCGTTTGTTTGTTTTG 3106
rat_BDNF        GAACGGGAGGGGTAGATTTCTGTTTGTTTTGTGTTTTATTTCGTTTGTTTGTTTGTTTTG 2964
mouse_BDNF      GAATGGGAGGGGTAGATTTCTGTTTGTTTTGTGTTTTATTTTGTTTGTTTGTTTGTTTTG 3010
                * * ***  ***** **** ***************** ***   * **************

human_BDNF      TTTTTTAGTTCCCACAGGGAGTAGAGATGGGGAAAGAATTCCTACAATATATATTCTGGC 3166
rat_BDNF        TTTTTTTAGTTCCACCCGGAGTAGGGATGGAGAAA--ATTTCTTCACTCTCCATTCTGGT 3022
mouse_BDNF      TTTTTT-AGTTCCACCCGGAGTAGGGATGGAGAAA--ATTTCTTCACTATCCATTCTGGT 3067
                ******   * ****  ******* ***** ****  *** ** ** * *  *******

human_BDNF      TGATAAAA-GATACATTTGTATGTTGTGAAGA-TGTTTGCAATATC-GATCAGATGACTA 3223
rat_BDNF        TGATAAAGCGTTACATTTGTATGTTGTAAAAAATGTTTGCAAAATCCAATCAGATGACTG 3082
mouse_BDNF      TGATAAAGCGTTACATTTGTATGTTGTAAAGA-TGTTTGCAAAATCCAATCAGATGACTG 3126
                *******  * **************** ** * ********* ***  ***********

human_BDNF      GAAAGTGAATAAAAATTAAGGCAACTGAACAAAAAAATGCTCACACTCCACATCCCGTGA 3283
rat_BDNF        GAAAACGAATAAAAATTAAGGCAACTGAATAAAA---TGCTCACACAACACTGCCCATGA 3139
mouse_BDNF      GAAAACAAATAAAAATTAAGGCAACTGAATAAAA---TGCTCACACTCCACTGCCCATGA 3183
                ****   ********************** ****   *********  ***  *** ***

human_BDNF      TGCACCTCCCAGGCCCC-----GCTCATTCTTTGGGCGTTGGTCAGAGTAAGCTGCTTTT 3338
rat_BDNF        TGTATCTCCCTGGTCCCCC--AGGTCACTCTTCTGGCATGGGTCAGGGAAAGCTGCTTTT 3197
mouse_BDNF      TGTATCTCCCTGGTCCCCCTCAGCTCACTCTTCTGGCATGGGTCAGGGAAAATTGCTTTT 3243
                ** * ***** ** ***     * *** ****  *** * ****** * **  *******

human_BDNF      GACGGAAGGACCTATGTTTGCTCAGAACACATTCTTTCCCCCCCTCCCCC---TCTGGTC 3395
rat_BDNF        ATTGGAAAGACCAGCATTTGTTTAAAGCACATTCTTTCCCTCCCTCCTCCCATTTTGGTC 3257
mouse_BDNF      ATTGGAAAGACCAGCATTTGTTCAAAGCATACTCTTTCCCTCCCTCCTCCCATTTTGGTC 3303
                   **** ****    **** * * * ** * ******** ****** **   * *****

human_BDNF      TCCTC----TTTGTTTTGTTTTAAGGAAGAAAAATCAGTTGCGCGTTCTGAAATATTTTA 3451
rat_BDNF        CCTTTCTTTTTTGTTTTGTTTTAAGAAAGAAAATTAAGTTGCGCGCTTTGAAATATTTTA 3317
mouse_BDNF      CCTTC--TTTTTGTTTTGTTTTAAGAAAGAAAATTAAGTTGCGCGCTTTAAAATATTTTA 3361
                 * *     **************** ******* * ********* * * **********

human_BDNF      CCACTGCTGTGAACAAGTGAACACATTGTGTCACATCATGACACTCGTATAAGCATGGAG 3511
rat_BDNF        TCACTGCTGTGAACAGATGAACAATGTGTGTCATTTCATGACACTC--------GTGGAA 3369
mouse_BDNF      CTACTGCTACAAACAGATGAACAATGTGTGTCATTTTATGACACTC--------ATGGAA 3413
                  ******   ****  ******   *******  * *********         ****

human_BDNF      AACAGTGATTTTTTTTTAGA-----ACAGAAAACAACAAAAAATAACCCCAAAATGAAGA 3566
rat_BDNF        AACAGTGATTTTTTTTTTATTTTTTGCCCTAAGGAGAAACAAGTAAGAATAACCGAAAAT 3429
mouse_BDNF      AACAGTGATTTTTTTTT--------ACCCTAAAGAAAAACAAATAAAAATAACCCAAAAT 3465
                *****************         *   **  *  ** ** ***    **    **

human_BDNF      TTATTTTTTATGAGGAGTGAACATTTGGGTAAATCATGGCTAAGCTTAAAAAAAACTCAT 3626
rat_BDNF        GTTCTTTTTTTTTAAAG---------GCATAAACAGTGGATAAGTT------------AT 3468
mouse_BDNF      ATTCTTTTTTTA-AAAG---------GCATAAATATTGGGTAAATT------------GT 3503
                 *  ***** *    **         *  ****   *** ***  *             *

human_BDNF      GGTGAGGCTTAACAATGTCTTGTAAGCAAAAGGTAGAGCCCTGTATCAACCCAGAAACAC 3686
rat_BDNF        AATATGGCCTAACAATGT-TTGCAGATAAAAGATA-----TTGCATACAGCCAGA--TAC 3520
mouse_BDNF      AATATGGCCTAACAGTGT-TTGCAGATAAAAGTTA-----TTGTATACACCCAGA--TAC 3555
                  *  *** ***** *** *** *   ***** **      ** **  * *****   **

human_BDNF      CTAGATCAGAACAGGAATCCACATTGCCAGTGACATGAGACTGAACAGCCAAATGGAGGC 3746
rat_BDNF        T------AGAGCAGGGATCCACACTGCCACTGAAATGCGACTGAATGGCCCTGTGGAGGC 3574
```

```
mouse_BDNF      TTAGATAAGAGCAGGGATCCACACTGCCATTGAAATAGGACTGAATGGCCCTGCGGAGGC 3615
                       *** **** ******* ***** *** **  *******  ***    ******

human_BDNF      TATGTGGAGTTGGCATTGCATTTACCGGCAGTGCGGGAGGAATTTCTGAGTGGCCATCCC 3806
rat_BDNF        TAAGTGGAGCTGACATACTATTTCCTGGCAGAGCAGGAGGAATTTCTGAGTGGCCATCCT 3634
mouse_BDNF      TAAGTGGAGCTGACATACTATTTCCTGGCAGTGCAGGAGGAATTTCTGAGTGGCCATCCT 3675
                ** ****** ** ***   **** * ***** ** ***********************

human_BDNF      AAGGTCTAG-GTGGAGGTGGGGCATGGTATTTGAGACATTCCAAAACGAAGGCCTCTGAA 3865
rat_BDNF        GAGGTCTAG-ATGGAGGTGGGGAATGGTACTTGAGACATTCCTAAAGGAAGGC-TCGGAA 3692
mouse_BDNF      AAGGTCTAGGATGGAGGTGGGGAATGGTACTTGAGACATTCCTAAAGGAAGGC-TCGGAA 3734
                 ********  *********** ****** ************ *** ****** ** ***

human_BDNF      GGACCCTTCAGAGGTGGCTCTGGAATGACATGTGTCAAGCTGCTTGGACCTCGTGCTTTA 3925
rat_BDNF        GCACCCTTCAGAGCAGGCTCTGGAATGA--TGTGTCAAGTTTCTTAGGCCTTCTGCTTTA 3750
mouse_BDNF      GCACCCTTCAGAGCAGGCTCTGGAATGA--TGTGTCAAGTTGCTTAGGCCTTCTGCTTTA 3792
                * ***********  *************  ********* * *** * ***  *******

human_BDNF      AGTGCCTACATTATCTAACTGTGCTCAAGAGGTTCTCGACTGGAGGACCACACTCAAGCC 3985
rat_BDNF        AGTGCCTACGTTACCTAACAGTGCTCAAGAGGTTCTCAATTGGAGAACCACACTCAAATC 3810
mouse_BDNF      AGTGCCTACATTACCTAACAGTGCTCAAGAGGTTCTCGATTGGAGAACCACACTCAAATC 3852
                ********* *** ***** **************** * ***** ***********  *

human_BDNF      GACTTATGCCCACCATCCCACCTCTGGATAATTTTGCATAAAATTGGATTAGCCTGGAGC 4045
rat_BDNF        CATTTATGGCCTCCATCCCATTT-TAAATAATTATGGATAAAGTTGGATTAACCTGGAGC 3869
mouse_BDNF      CATTTATAGCCTCCATCCCATTTCTAAATAATTGTGTATAAAGTTGGATTAACCTGGAGC 3912
                 * ****  ** ********  * *  ****** ** ***** ******** ********

human_BDNF      AGGTTGGGAGCCAAATGTGGCATTTGTGATCATGAGATTGATGCAATGAGATAGAAGATG 4105
rat_BDNF        AGCTTTGGATCCAAATATGGCATA-GCAGTGATGCTATCAGTGCAGCATGATGGGAAATG 3928
mouse_BDNF      AACTTTGGATCCAAATATGGCACA-GCAATAATGATATTAATGCAGCATGATGGGAAATG 3971
                *  ** *** ****** *****   *   * ***  **   ****    *** * * ***

human_BDNF      TTTGCTACCTGAACACTTA--TTGCTTTGAAACTAGACTTGAGGAAACCAGGGTTTATCT 4163
rat_BDNF        TTTGCTGTGAAGAGACTTAACTTTCTTTGCGCTTAGACTTCAGGAAGCCTAGGTTTTATT 3988
mouse_BDNF      TTTGCTGTGAAGAGAATTGATTTGCTTTGAGCTTAGACTTCAGGAAGCCTAGGTTTTTTA 4031
                ******      * * **   ** *****    ******* ***** **  *****

human_BDNF      TTTGAGAACTTTTGGTAAGGGAAAAGGGAACAGGAAAAGAAACCCCAAACTCAGGCCGAA 4223
rat_BDNF        TATTTATTTTTTTG--AGACATTTTGGTGAAAGGAAAAAA------GAAAGAAG--AAAA 4038
mouse_BDNF      TTTTTTTATTTTTG--AGACATTTTGGTAAAAGGAAAAAAAG----AAAACAAA--CAAA 4083
                * *      *****  *        **  * ******* *       **   *     **

human_BDNF      TGATCAAGGGGACCCATAGGAAATCTTGTCCAGAGACAAGACTTCGGGAAGGTGTCTGG- 4282
rat_BDNF        CAAACAAACAAAACCAGAAAAAGC---ACCAAAACTTAGGCAGAATGAGCAATGTCTGTC 4095
mouse_BDNF      CAAACAAACAAAACCAGAAAAAGC---ATCAAAACTCAGGCAGAATGAGCAATGTCTGA- 4139
                  * ***    * *** *  **        * * *   * *      *     ******

human_BDNF      ----ACATTCAGAACACCAAGACTTG--AAGGTGCCTTGCTCAATGGAAGAGGCCAGGAC 4336
rat_BDNF        TGTAAGGGCTAGAATGACAAGGCATAGGAAGGTGCTTT-CACTGTGAAAGAGACAAGAAC 4154
mouse_BDNF      ---AAGGGCTAGAAAAACAAGACATAGCAAGGTGCTTT-CACTGTGAAAGAGACAAGAAC 4195
                    *     ****   **** * *  ******* ** * *  ** ***** * ** **

human_BDNF      AGAGCTGACAAAATTTTGCTCCCCAGTGAAGGCCACAGCAACCTTCTGCCCATCCTGTCT 4396
rat_BDNF        ACAG--GAGGAAATACTGCTTA--AGTGAAGAGCACAGAAAGCTCCTGATAGTTCTGTCC 4210
mouse_BDNF      ACAG--GAGGAAATATTGCTTC--AGTGAAGAGCACAGACGGCTCCTGCCA--------- 4242
                * **  **  ****  ****    *******  *****     ** ***

human_BDNF      GTTCATGGAGAGGGTCCCTGCCTCACCTCTGCCATTTTGGGTTAGGAGAAGTCAAGTTGG 4456
rat_BDNF        ATTCAGCACAAGGGTCCCTTCTACACTT-TACCTCTTGGGGTTAGGAGAAGTCAAGCTGG 4269
mouse_BDNF      ATTTATTACAAGAGTCCCGTCTGTACTT-TACCCTTTGGGGTTAG---AAGTCAAGTTGG 4298
                 ** *     ** *****  *   ** * * **  ** *******   ******** ***

human_BDNF      GAGCCTGAAATAGTGGTTCTTGGAAAAATGGATCCCCAGTGAAAACTAGAGCTCTAAGCC 4516
rat_BDNF        AAGCCTGAATGAATGGCCCCAATGAGAACTAGT-----GTTAAGCCCATTTCCCTAG--- 4321
mouse_BDNF      AAGCCTGAATGAATGGACCCAATGAGAACTAGT-----GTTAAGCCCATTTCCCTAG--- 4350
                 ********  * ***  *     * **     *      ** **  * *   * ***
```

```
human_BDNF      CATTCAGCCCATTTCACACCTGAAAATGTTAGTGATCAC-CACTTGGACC----AGCATC 4571
rat_BDNF        --TGAGGTTTTCCGCCAGCGCGAATGTGTTAGTGGTTACCTGACTGGGCTCCTGGGCATC 4379
mouse_BDNF      --TCAGGTTTTTTTCAAGCGTGAATGTGTTAGTGGTTACTCTCCTGGGTTCCTGAGCATC 4408
                  *   *       *   *  ***  ******** * **      ***        *****

human_BDNF      CTTAAGTATCAGAAAGCCCCAAGCAATTGCTGCATCTTAGTAGGGTGAGGGATAAGCAAA 4631
rat_BDNF        AGAAAAA--------GAGGCAAACAATTGCTTCATCTTAGGAGTGGAAAGGGTGAAACAA 4431
mouse_BDNF      AGAAAAAAAAAAAAAGAGGCAAACAATCGCTTCATCTTAGGAGTGGAAAGGA--AACAGA 4466
                   **           *   *** **** *** ******** ** *  * **    *    *

human_BDNF      AGAGGATGTTCACCATAACCCAGG-AATGAAGATACCATCAGCAAAGAATTTCAATTT-G 4689
rat_BDNF        AGTGGCTGTCCACTGTGACTCAGGGAGTGAAGATACCATCAGCAAATAGTTTCTTTTT-G 4490
mouse_BDNF      AGTGGACGTCCGCTGTGACTCAGGGAGTGAAGATACCATCAGCAAATAGTTTCTTTTTTG 4526
                ** **  ** * *  * ** **** * ****************** * ****  *** *

human_BDNF      TTCAGTCTTTCATTTAGAGCTA----GTCTTTCACAGTACCATCTGAATACCTCTTTGAA 4745
rat_BDNF        TTCAACTGTTCCTTTAGAACTAGTCTGTCTTCTGGAGTCCCA-CTGAATCCCCTGTTTTT 4549
mouse_BDNF      TTCATTCGTTCCTTTCGAGTTAGCCTGTCTTTTGGAATACCA-CTGAATATGCTGTTTTT 4585
                ****    *** *** **  **     *****     * * *** ******       **

human_BDNF      AGAAGGAAGACTTTACGTAGTGTAGATTTGTTTTGTGTTGTTTGAAAATATTA-TCTTTG 4804
rat_BDNF        ----GGAAGACTTCACGTAGCCTAGATT-GTTTTGTGCCGTTTGACAACATTAATCTCTG 4604
mouse_BDNF      ----GAAAGACTTCATGTAGCATAGATT-GTTTTGTGCCGTTTACCAA-ATTAACCTTTG 4639
                    * ******* * ****  ****** ********  ****   ** ****  ** **

human_BDNF      TAATTATTTTT-AATATGT-AAGGAATGCTTGGAATATCTGCTATATGTCAACTTTATGC 4862
rat_BDNF        TCATCATTTTT-AACCTATTAAGGAATGCTTTGAATATCTGCTATATGCTAACTTTTTGC 4663
mouse_BDNF      TCATCGTTTTTTAACCTATTCAGGAATGCTTGGAATATCTGCTCTATGTTAACTTTTTGC 4699
                * **  ***** **   * *  ********** *********** ****  ****** ***

human_BDNF      AGCTTCCTTTTGAGGGACAAATTTAAAACAAACAAC----CCCCCATCACAAACTTAAAG 4918
rat_BDNF        AGCTTCATTCTGAGAGACGTTAGTCAAACAAATAAAAGGAGCCCCATCACAATCTCACGG 4723
mouse_BDNF      AGCTTCATTCTGAGAGACATTAGTCAAACAAACAAAAGGATCCCCATCACAATCTTACAG 4759
                ****** ** **** ***      * ******* **      *********** ** *  *

human_BDNF      GATTGCAAGGGCCAGATCTGTTAAGTGGTTTCATAGGAGACACATCCAGCAATTGTGTGG 4978
rat_BDNF        TATTCGAAGGGCCAGGTCGATTAGGTGGCTTCATAGGAGACCC--TCCGCAACTGTGTGG 4781
mouse_BDNF      TACTGCAAGGGCCAGGTCTGTTAAGCGGCTTCACAGGAGACA---TCAGCAATTGTGTGG 4816
                 * *  ********* **   *** * ** **** *******      * **** *******

human_BDNF      TCAGTGG----CTCTTTTACCCAATAAGATACATCACAGTCACATGCTTGATGGTTTATG 5034
rat_BDNF        TCAGTGGCTGGCTCTCATACCCACTAAGATACATCATAGCTCCATG-TCGGTGGTTTATG 4840
mouse_BDNF      TCAGTGGCTGGCTCTCTTACCCACTAAGATACATCATAGCTACATG-TTGGTGGTTTATG 4875
                *******    ****  ****** ************ **    **** * * *********

human_BDNF      TTGACCTAAGATTTATTTTGTTAAAATCTCT-CTCTGTTG-TGTTCGTTCT--------- 5083
rat_BDNF        TTGACCTGAGATTGATTT-GTTAAAATCTCTCCTCTGTTTCTGTTCGTTCTGTTTCCGTC 4899
mouse_BDNF      TTGACCTGAGATTTATTT-GTTAAAATCTCTTCTTCGTTTCTGTTCGTTCTGGTTCTGTT 4934
                ******* ***** **** ************ **  ***  **********

human_BDNF      -TGTTCTGTTTTGTTTTGTTTTT----TAAAGTCTTGCTGTGGTCTCTTTGTGGCAGAAG 5138
rat_BDNF        CTGTTCTGTTCTGTTCTGTTCTG-----AAAGTCTTGCTGTGGTCTCTTTTTGGCAGAAG 4954
mouse_BDNF      CTGTTCTGTTCTGTTCTGTTTTGGTTTTAAAGTCTTGCTGTGGTCTCTTGTTGGCAGAAA 4994
                 ********* **** **** *      *********************  ********

human_BDNF      TGTTTCATGCATGGCAGCAGGCCTGTTGCTTTTTTATGGCGATTCCCATTGAAAATGTAA 5198
rat_BDNF        TGTTTCATGCATGGCAGCAGGCCTGATGCTTTTT-ATAGTGATTCCCATTGAAACTGTAA 5013
mouse_BDNF      TGTTTTATGCATGGCAGCAGGCCTGTTGCTTTTTTATAGTGATTCCCATTGAAACTGTAA 5054
                ***** ******************* ******** ** * ************** *****

human_BDNF      GTAAATGTCTGTGGCCTTGTTCTCTCTATGGTAAAGATATTATTCACCATGTAAAACAAA 5258
rat_BDNF        GTAAATGTCTGTGGCCTTGTTCTCTCTATGGTAAAGATATTATTCACCATGTAAAACAAG 5073
mouse_BDNF      GTAAATGTCTGTGGCCTTGTTCTCTCTATGGTAAAGATATTATTCACCATGTAAAACAAG 5114
                ***********************************************************

human_BDNF      AAACAATATTTATTGTATTTTAGTATATTTATATAATTATGTTATTGAAAAAAATTGGCA 5318
rat_BDNF        AAAAA-TATTTATTGTATTTTAGTATATTTATATAATTATGTTATTGAAAAAAATTGGCA 5132
mouse_BDNF      AAAAAATATTTATTGTATTTTAGTATATTTATATAATTATGTTATTGAAAAAA-TTGGCA 5173
```

```
                     *** * *********************************************** ******

human_BDNF           TTAAAACTTAACCGCATCAGAA-CCTATTGTAAATACAAGTTCTATTTAAGTGTACTAAT 5377
rat_BDNF             TTAAAACTTAACCACATCAGAAGCCTATTGTAAATACAGGTTCTATTTAAGTGTACCAAT 5192
mouse_BDNF           TTAAAACTTAACCACATCAGAAGCCTATTGTAAATACAGGTTCTATTTAAGTGTACCAAT 5233
                     ************* ******** *************** ***************** ***

human_BDNF           TAACATATAATATATGTTTTAAATATAGAATTTTTAATGTTTTTAAATATATTTTCAAAG 5437
rat_BDNF             TAACATATAATATATGTTTTAAATATAGAATTTTTAATGTTTTTAAATATATTTTCAAAG 5252
mouse_BDNF           TAACATATAATATATGTTTTAAATATAGAATTTTTAATGTTTTTAAATATATTTTCAAAG 5293
                     ************************************************************

human_BDNF           TACATAAAATCTGGGGGTTCTGGGTTTTTTTCTCTTTACTGTAAAACAAACATGAAAACG 5497
rat_BDNF             TACATAAAATCTGGGGGTTCTGGGTTTTTT-CTCTTCACTGTAAAATA----TGAAAATG 5307
mouse_BDNF           TACATAAAGTCTGGGGGTTCTGGGTTTTTT-CTCTTCACTGTAAAATA----TGAAAATG 5348
                     ******** ********************* ***** ********* *    ****** *

human_BDNF           TGTTTTATTATAAATACGTGTGTTCCTTGCTTT 5530
rat_BDNF             TGTTTTATTATAAATACATGTGTTCCTTGCTTT 5340
mouse_BDNF           TGTTTCATTATAAATACATGTGTTCCTTGCTTT 5381
                     ***** *********** ***************
```
